# Empagliflozin enhances metabolic efficiency and improves left ventricular hypertrophy in a hypertrophic cardiomyopathy mouse model

**DOI:** 10.1093/eurheartj/ehaf324

**Published:** 2025-05-21

**Authors:** Tomas Baka, Jarrod Moore, Fuzhong Qin, Salva R Yurista, Aifeng Zhang, Huamei He, Jordan M Chambers, Dominique Croteau, Raghuveera K Goel, Hunter Smith, Miranda C Wang, Christopher S Chen, Ion A Hobai, Martina Rombaldova, Ondrej Kuda, Jil C Tardiff, James A Balschi, David R Pimentel, Christine E Seidman, Jonathan G Seidman, Andrew Emili, Wilson S Colucci, Ivan Luptak

**Affiliations:** Myocardial Biology Unit, Boston University School of Medicine, 650 Albany Street, Evans Biomed Research Ctr (room 704B), Boston, MA 02118, USA; Center for Network Systems Biology, Department of Biochemistry, Boston University School of Medicine, Boston, MA, USA; Myocardial Biology Unit, Boston University School of Medicine, 650 Albany Street, Evans Biomed Research Ctr (room 704B), Boston, MA 02118, USA; Myocardial Biology Unit, Boston University School of Medicine, 650 Albany Street, Evans Biomed Research Ctr (room 704B), Boston, MA 02118, USA; Myocardial Biology Unit, Boston University School of Medicine, 650 Albany Street, Evans Biomed Research Ctr (room 704B), Boston, MA 02118, USA; Physiological NMR Core Laboratory, Brigham and Women’s Hospital, Harvard Medical School, Boston, MA, USA; Myocardial Biology Unit, Boston University School of Medicine, 650 Albany Street, Evans Biomed Research Ctr (room 704B), Boston, MA 02118, USA; Myocardial Biology Unit, Boston University School of Medicine, 650 Albany Street, Evans Biomed Research Ctr (room 704B), Boston, MA 02118, USA; Center for Network Systems Biology, Department of Biochemistry, Boston University School of Medicine, Boston, MA, USA; Myocardial Biology Unit, Boston University School of Medicine, 650 Albany Street, Evans Biomed Research Ctr (room 704B), Boston, MA 02118, USA; Department of Biomedical Engineering, Boston University, Boston, MA, USA; Wyss Institute for Biologically Inspired Engineering, Harvard University, Boston, MA, USA; Harvard-MIT Program in Health Sciences and Technology, Institute for Medical Engineering and Science, Massachusetts Institute of Technology, Cambridge, MA, USA; Department of Biomedical Engineering, Boston University, Boston, MA, USA; Wyss Institute for Biologically Inspired Engineering, Harvard University, Boston, MA, USA; Department of Anesthesia, Critical Care and Pain Medicine, Massachusetts General Hospital, Boston, MA, USA; Laboratory of Metabolism of Bioactive Lipids, Institute of Physiology of the Czech Academy of Sciences, Prague, Czech Republic; Laboratory of Metabolism of Bioactive Lipids, Institute of Physiology of the Czech Academy of Sciences, Prague, Czech Republic; Department of Biomedical Engineering, University of Arizona, Tucson, AZ, USA; Department of Medicine, University of Arizona, Tucson, AZ, USA; Physiological NMR Core Laboratory, Brigham and Women’s Hospital, Harvard Medical School, Boston, MA, USA; Myocardial Biology Unit, Boston University School of Medicine, 650 Albany Street, Evans Biomed Research Ctr (room 704B), Boston, MA 02118, USA; Department of Genetics, Harvard Medical School, Boston, MA, USA; Division of Cardiovascular Medicine, Brigham and Women’s Hospital, Boston, MA, USA; Howard Hughes Medical Institute, Chevy Chase, MD, USA; Department of Genetics, Harvard Medical School, Boston, MA, USA; Center for Network Systems Biology, Department of Biochemistry, Boston University School of Medicine, Boston, MA, USA; Myocardial Biology Unit, Boston University School of Medicine, 650 Albany Street, Evans Biomed Research Ctr (room 704B), Boston, MA 02118, USA; Myocardial Biology Unit, Boston University School of Medicine, 650 Albany Street, Evans Biomed Research Ctr (room 704B), Boston, MA 02118, USA

**Keywords:** Hypertrophic cardiomyopathy, SGLT2 inhibition, Cardiac energetics, Cardiac metabolism, Uncoupled glycolysis, Branched-chain amino acids, Metabolic reprogramming, mTOR

## Abstract

**Background and Aims:**

Hypertrophic cardiomyopathy (HCM) is a genetic cardiac disorder characterized by left ventricular hypertrophy (LVH), diastolic dysfunction, and impaired metabolic efficiency. This study investigates the therapeutic potential of the sodium–glucose cotransporter 2 inhibitor (SGLT2i) empagliflozin (EMPA) in ameliorating these pathological features in a mouse model carrying the myosin R403Q mutation.

**Methods:**

Male mice harbouring the R403Q mutation were treated with EMPA for 16 weeks. Multi-nuclear magnetic resonance spectroscopy (^31^P, ^13^C, and ^23^Na MRS), echocardiography, transcriptomic, proteomic, and phosphoproteomic profiling were utilized to assess metabolic, structural, and functional changes.

**Results:**

Empagliflozin facilitated the coupling of glycolysis with glucose oxidation and normalized elevated intracellular sodium levels. Treatment resulted in a significant reduction in LVH and myocardial fibrosis as evidenced by echocardiography and histopathology. These structural improvements correlated with enhancements in mitochondrial adenosine triphosphate (ATP) synthesis, fatty acid oxidation, and branched-chain amino acid catabolism. Furthermore, EMPA improved left ventricular diastolic function and contractile reserve, underscored by improved ATP production and reduced energy cost of contraction. Notably, these benefits were linked to down-regulation of the mammalian target of rapamycin signalling pathway and normalization of myocardial substrate metabolic fluxes.

**Conclusions:**

Empagliflozin significantly mitigates structural and metabolic dysfunctions in a mouse model of HCM, underscoring its potential as a therapeutic agent for managing this condition. These findings suggest broader applicability of SGLT2i in cardiovascular diseases, including those due to myocardial-specific mutations, warranting further clinical investigation.

## Structured Graphical Abstract

**Structured Graphical Abstract:**
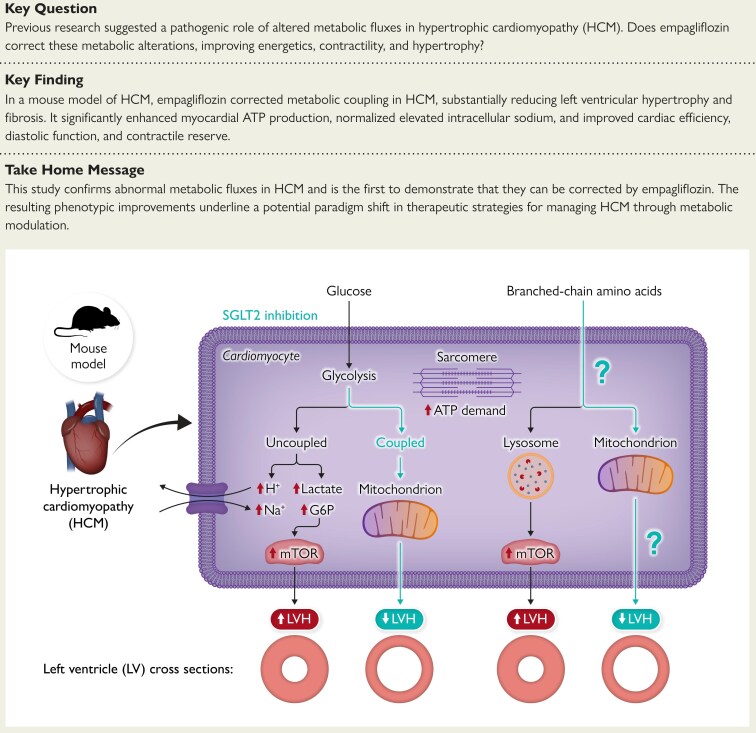

**See the editorial comment for this article ‘Empagliflozin to energize the heart of patients with hypertrophic cardiomyopathy’, by J. van der Velden, https://doi.org/10.1093/eurheartj/ehaf395.**

Translational Perspective Recent advancements in preventing sudden death have shifted the focus to heart failure (HF) as the primary cause of morbidity and mortality in patients with hypertrophic cardiomyopathy (HCM), highlighting an unmet clinical need. This study suggests that sodium–glucose cotransporter 2 inhibitors could offer broad benefits in HCM, by correcting metabolic dysregulation associated with reducing left ventricular hypertrophy, fibrosis, and contractile dysfunction. These findings support their potential to improve cardiac function and delay the progression to HF. Moreover, this study contributes to the evidence supporting the use of myocardial metabolic modulation in cardiovascular diseases.

## Introduction

Hypertrophic cardiomyopathy (HCM) is a hereditary cardiac disorder caused by mutations in several sarcomere genes, integral to the heart's contractile machinery. With an estimated prevalence of 1:200, the clinical presentation of HCM spans a spectrum from asymptomatic stages to severe complications such as heart failure (HF) and life-threatening arrhythmias, highlighting its significance as a public health burden. While strategies for sudden death prevention have been successful, HF continues to be the main contributor to morbidity and mortality in HCM patients.^1,2^

At the cellular level, HCM mutations lead to a state of hypercontractility and an energetically inefficient sarcomere, leading to a several-fold increase in adenosine triphosphate (ATP) consumption.^3,4^ This culminates in left ventricular (LV) hypertrophy (LVH) and diastolic dysfunction.^3^ The progression to an advanced phenotype, characterized by profound mitochondrial dysfunction, reduced capacity for increased systolic workload during physical exertion, and myocardial fibrosis, is not fully elucidated.^5–8^ As has been shown in pressure overload, there is a preferential reliance on glycolysis over fatty acid oxidation (FAO) in pathological LVH, without a corresponding increase in glucose oxidation, leading to an accumulation of glycolytic intermediates.^9^ Similarly, increased myocardial glucose uptake^10^ and compromised energetics, as evidenced by reduced phosphocreatine-to-ATP ratio (PCr/ATP),^6,11^ have been noted to precede LVH in patients with HCM. Our recent multi-omic investigations have corroborated these findings, demonstrating an early accumulation of glycolytic intermediates and a decline in FAO enzymes in advanced HCM.^12^

Taken together, these observations suggest that uncoupling of glycolysis from glucose oxidation may be pathogenic in HCM. The accumulation of glycolytic byproducts, including pyruvate, lactate, and protons, may exert cardiotoxic effects leading to elevated intracellular sodium,^13,14^ decreased FAO, and mitochondrial damage in advanced HCM. Elevated intracellular sodium, in turn, can impair intracellular calcium handling, a mechanism implicated in HCM pathogenesis.^15,16^ Importantly, while similar substrate changes have been suggested by *ex vivo* metabolomic analysis,^12^ to our best knowledge, no direct measurements of substrate oxidation fluxes have been previously performed in beating HCM hearts.

In this context, sodium–glucose cotransporter 2 inhibitors (SGLT2i), such as empagliflozin (EMPA), have emerged as promising therapeutic agents, demonstrating substantial benefits on cardiovascular morbidity and mortality in HF patients, irrespective of diabetic status.^17^ Our recent findings from diabetic cardiomyopathy suggest that SGLT2 inhibition has the potential to counter LVH, increase FAO, and lower myocardial intracellular sodium,^18,19^ thereby offering a compelling rationale for exploring their utility in HCM management. This is further supported by SGLT2i-related improvements in diastolic function and functional capacity^20^ as well as all-cause mortality, hospitalizations, and cardiovascular symptoms in clinical studies encompassing diabetic and non-diabetic HCM patients.^21^

Here, we hypothesized that treatment with EMPA would improve cardiac energetics, contractile function and structure in HCM. To test this hypothesis, we studied mice carrying the myosin R403Q mutation treated with EMPA for 16 weeks. Through the application of multi-nuclear magnetic resonance spectroscopy (^31^P, ^13^C, and ^23^Na MRS), alongside transcriptomic, proteomic, and phosphoproteomic profiling, we determined that EMPA treatment facilitates the coupling of glycolysis with glucose oxidation and normalizes the elevated intracellular sodium levels observed in HCM myocardium. Consistent with prior literature, EMPA normalized myocardial FAO^18,22^ and, unexpectedly, branched-chain amino acid (BCAA) catabolism,^23^ which was associated with reduced mammalian target of rapamycin (mTOR) activation. These metabolic corrections were accompanied by the attenuation of LVH and fibrosis, improved energetics, diastolic function, and contractile reserve in HCM hearts.

## Methods

**Figure 1:**
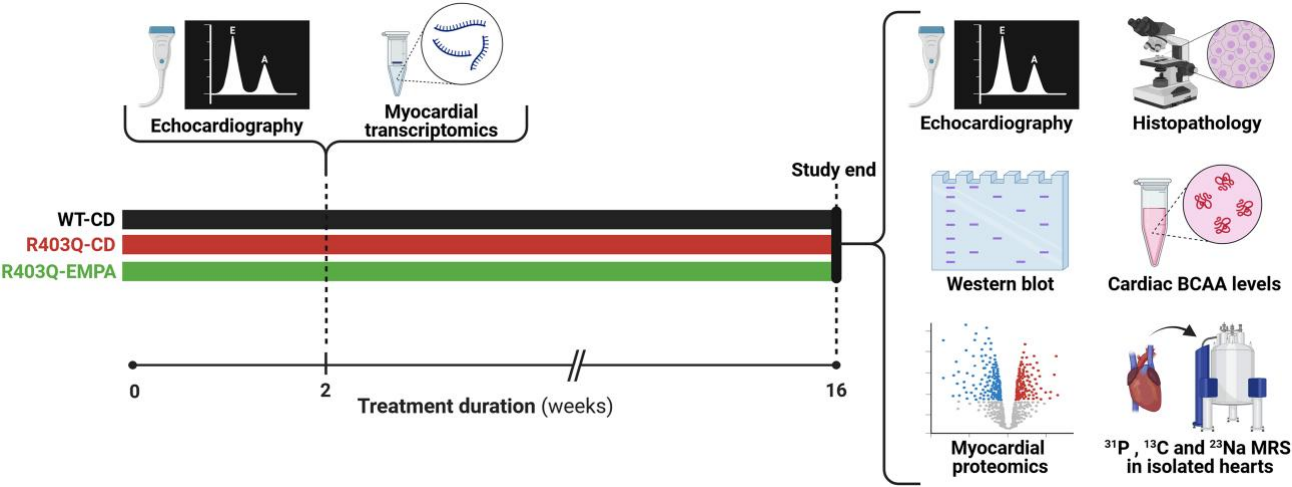
Study protocol. Mice harbouring R403Q mutation in cardiac myosin heavy chain or their wild-type littermates were randomized by body weight into three groups: (i) wild-type-control diet (WT-CD); (ii) R403Q-CD; and (iii) R403Q-EMPA. Nine-to-10-weeks-old mice were started on a control diet or the same diet enriched with empagliflozin. After 2 weeks of treatment echocardiography was performed and subset of mice was sacrificed for myocardial RNA sequencing analysis. At the end of the 16-week treatment period, mice underwent echocardiography and heart isolation followed by ^31^P, ^13^C, and ^23^Na NMR spectroscopy. Hearts from a separate set of animals were used for histology, western blot analysis, cardiac branched-chain amino acid levels measurement, and both proteomics and phosphoproteomics assessments. Created with BioRender.com

Nine-to-10-week-old male mice harbouring R403Q mutation in cardiac myosin heavy chain or wild-type (WT) littermates were fed *ad libitum* a control diet (CD; 10% kcal lard, 0% sucrose; product No. D09071703; Research Diets, New Brunswick, NJ, USA) or the same diet enriched with EMPA (0.15 mg/g of diet; Boehringer Ingelheim, Ingelheim am Rhein, Germany) for 16 weeks. This dose of EMPA is equivalent to ∼15 mg/kg body weight as described by Verma *et al*.^24^ Mice harbouring R403Q mutation in cardiac myosin heavy chain were kindly provided by Drs Christine E. Seidman and Jonathan G. Seidman (Harvard Medical School, Boston, MA, USA).^3,25,26^ Mice were randomized by body weight and divided into three groups: (i) WT-CD; (ii) R403Q-CD; and (iii) R403Q-EMPA. Mice were housed under standard laboratory conditions with controlled temperature, humidity, and light. The protocol was approved by the Institutional Animal Care and Use Committee at Boston University School of Medicine and Brigham and Women's Hospital. The study protocol is depicted in *Figure 1*.

Echocardiography,^18,27^ LV contractile function, and high-energy phosphates measurement by ^31^P NMR spectroscopy,^18,19,28,29^  ^13^C substrate oxidation flux by ^13^C NMR^30–32^ and intracellular sodium [Na^+^]*_i_* assessment by ^23^Na NMR in isolated beating hearts,^19^ cardiac histology,^18,28^ and western blot analysis,^33,34^ BCAA levels measurement, transcriptomics,^18^ proteomics, and phosphoproteomics^12^ were performed as previously described. Supplementary animal studies, detailed methods and dataset deposition information are available in the Supplementary data online. Means, SEM, and *N* for each figure are provided in Supplementary data online, *Table S5*.

## Results

### Empagliflozin modulates pathways associated with substrate metabolism, energetics, and hypertrophy in R403Q hearts

Transcriptomic and proteomic/phosphoproteomic analyses were conducted to evaluate the effects of EMPA on R403Q hearts. Echocardiography in 12-week-old mice revealed diastolic LV dysfunction without LVH (see Supplementary data online, *Figure S1*). Transcriptomic analysis of 12-week-old R403Q hearts, prior to the onset of LVH, revealed: (i) up-regulation of pathways related to hypertrophy and remodelling, including mammalian target of rapamycin complex 1 (mTORC1) signalling and collagen formation; (ii) down-regulation of energy metabolism pathways including pyruvate dehydrogenase (PDH) complex, FAO, tricarboxylic acid (TCA) cycle and mitochondrial electron transport, (iii) down-regulation of DNA metabolism and up-regulation of ATPase-coupled ion transport; (iv) down-regulation of insulin and up-regulation of glucagon related pathways; (v) down-regulation of BCAA catabolism; and (vi) pathological changes in pathways related to cardiac muscle contraction, relaxation, cardiac cell development, and action potential formation. Remarkably, a brief (2-week) EMPA treatment counteracted these gene reprogramming changes (*Table 1* and Supplementary data online, *Table S1*). Furthermore, single-nucleus RNA sequencing corroborated these findings by demonstrating fibroblast activation^35^ in untreated R403Q hearts (see Supplementary data online, *Figures S2–S4*).

**Table 1:** Cardiac RNA sequencing analysis conducted on 12-week-old mice prior to the development of left ventricular hypertrophy

Keyword	Group	Gene set name	R403Q-CD vs WT-CD			R403Q-EMPA vs R403Q-CD		
			NES	*P*	FDR *q*	NES	*P*	FDR *q*
Fatty acid	C5 GO biological process	FATTY ACID BETA OXIDATION	−2.26	<.0001	***0.0007***	2.30	<.0001	***0.0046***
	C2 reactome pathway	MITOCHONDRIAL FATTY ACID BETA OXIDATION	−2.24	<.0001	***0.0008***	2.09	<.0001	***0.0292***
	C5 GO biological process	FATTY ACID CATABOLIC PROCESS	−2.15	<.0001	***0.0030***	2.12	<.0001	***0.0268***
	C2 WikiPathways pathway	FATTY ACID BETAOXIDATION	−2.01	<.0001	***0.0119***	2.38	<.0001	***0.0023***
	C2 KEGG pathway	FATTY ACID METABOLISM	−2.01	<.0001	***0.0120***	1.99	<.0001	***0.0468***
	C5 GO biological process	REGULATION OF FATTY ACID OXIDATION	−1.50	.0286	***0.1402***	2.01	<.0001	***0.0422***
	C5 GO biological process	REGULATION OF FATTY ACID BETA OXIDATION	−1.45	.0687	***0.1661***	2.10	<.0001	***0.0268***
Collagen	C2 reactome pathway	COLLAGEN DEGRADATION	2.42	<.0001	***<0.0001***	−1.93	<.0001	***0.0410***
	C2 reactome pathway	ASSEMBLY OF COLLAGEN FIBRILS AND OTHER MULTIMERIC STRUCTURES	2.35	<.0001	***<0.0001***	−1.99	<.0001	***0.0237***
	C5 GO cellular component	COLLAGEN CONTAINING EXTRACELLULAR MATRIX	2.34	<.0001	***<0.0001***	−1.73	<.0001	***0.1136***
	C5 GO cellular component	COLLAGEN TRIMER	2.31	<.0001	***0.0001***	−2.28	<.0001	***0.0002***
	C2 reactome pathway	COLLAGEN FORMATION	2.28	<.0001	***0.0001***	−2.25	<.0001	***0.0014***
	C2 reactome pathway	COLLAGEN CHAIN TRIMERIZATION	2.27	<.0001	***0.0001***	−2.36	<.0001	***0.0000***
	C5 GO cellular component	COMPLEX OF COLLAGEN TRIMERS	2.13	<.0001	***0.0004***	−2.10	<.0001	***0.0072***
	C2 reactome pathway	COLLAGEN BIOSYNTHESIS AND MODIFYING ENZYMES	2.09	<.0001	***0.0007***	−2.34	<.0001	***0.0000***
	C5 GO biological process	COLLAGEN METABOLIC PROCESS	2.03	<.0001	***0.0014***	−1.52	<.0001	***0.2422***
	C2 reactome pathway	CROSSLINKING OF COLLAGEN FIBRILS	1.90	.0038	***0.0052***	−1.74	<.0001	***0.1126***
	C5 GO biological process	REGULATION OF COLLAGEN METABOLIC PROCESS	1.71	.0036	***0.0249***	−1.61	<.0001	***0.1838***
BCAA	C2 reactome pathway	BRANCHED CHAIN AMINO ACID CATABOLISM	−2.47	<.0001	***<0.0001***	1.57	.0272	***0.2125***
Hypertrophy	C2 WikiPathways pathway	HYPERTROPHY MODEL	1.72	.0112	***0.0240***	−1.85	.0036	***0.0646***
Insulin	C5 GO biological process	REGULATION OF INSULIN LIKE GROWTH FACTOR RECEPTOR SIGNALING PATHWAY	1.97	<.0001	***0.0027***	−1.66	.0090	***0.1496***
PDH	C2 reactome pathway	REGULATION OF PYRUVATE DEHYDROGENASE PDH COMPLEX	−1.97	<.0001	***0.0164***	1.49	.0624	***0.2414***
	C2 WikiPathways pathway	TCA CYCLE AND DEFICIENCY OF PYRUVATE DEHYDROGENASE COMPLEX PDHC	−2.45	<.0001	***0.0001***	1.57	.0281	***0.2105***
Misc.	C5 GO biological process	CARDIAC CELL DEVELOPMENT	−1.93	<.0001	***0.0238***	1.79	<.0001	***0.1164***
	C5 GO biological process	VENTRICULAR CARDIAC MUSCLE CELL ACTION POTENTIAL	−2.16	<.0001	***0.0029***	1.50	.0275	***0.2376***

R403Q hearts showed: (i) up-regulation of pathways related to hypertrophy and remodelling, collagen formation and metabolism; (ii) down-regulation of pathways associated with fatty acid oxidation; (iii) pathological regulation of energetic metabolism pathways, characterized by the down-regulation of the TCA cycle and the PDH complex; (iv) dysregulation of metabolic signalling pathways, evident from down-regulation of insulin signalling; (v) down-regulation of BCAA catabolism; and (vi) pathological modulation of pathways related to cardiac muscle contraction and relaxation, cardiac cell development, and action potential formation. Differential expression was assessed using the Wald test implemented in the DESeq2 R package. Sample sizes were *n* = 5 for WT-CD, *n* = 7 for R403Q-CD, and *n* = 4 for R403Q-EMPA. Gene sets were obtained from the Molecular Signatures Database (MSigDB), version 7.5.1. False discovery rate (FDR) corrected *P*-values (FDRq) and nominal *P*-values (*P*) of each gene set are reported. An FDRq < 0.05 was considered significant.

**Figure 2:**
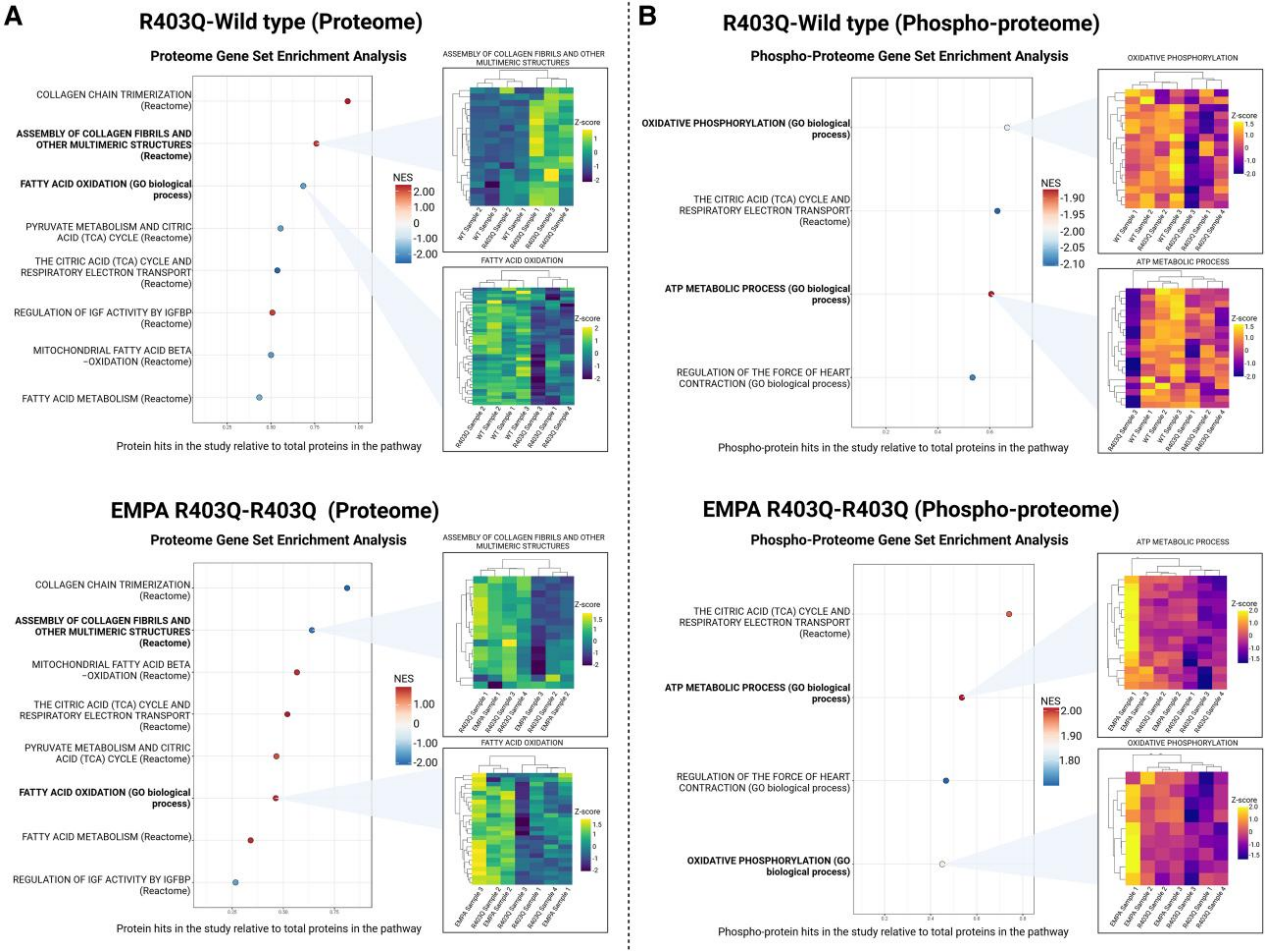
Cardiac proteomic and phosphoproteomic analysis performed in 25-week-old mice after 16 weeks of treatment. Cardiac proteomics showed (*A*) down-regulation of pathways associated with fatty acid oxidation and metabolism, pyruvate metabolism, and tricarboxylic acid and respiratory electron transport in the R403Q mice; up-regulation of pathways associated with collagen formation and the regulation of insulin-like growth factor activity in the R403Q mice. Treatment with empagliflozin countered these changes. Cardiac phosphoproteomics showed (*B*) down-regulation of pathways associated with oxidative phosphorylation, citric acid cycle and respiratory electron transport, adenosine triphosphate metabolic process, and regulation of the force of heart contraction. Treatment with empagliflozin countered these changes. The *n* = 3 (WT-CD), 4 (R403Q-CD), and 3 (R403Q-EMPA). Proteome and phosphor-proteome gene set enrichment analyses depict top differential pathways and select heatmaps with hierarchically clustered (Euclidean distance) profiles of corresponding annotated protein components. Pathways with FDR <0.05 in the R403Q-CD comparison was considered significant. Differential expression of pathways was assessed via a normalized enrichment score, in which a positive normalized enrichment score represents up-regulation and negative normalized enrichment score down-regulation in the first comparison group (e.g. positive normalized enrichment score in the plot comparing R403Q-WT signifies up-regulated in R403Q). Volcano plots are shown in Supplementary data online, *Figure S2*

Proteomic and phosphoproteomic analyses at the end of the study at 25-weeks of age further validated the transcriptomics findings. Compared with WT mice, untreated R403Q mice exhibited decreased enrichment of pathways related to pyruvate metabolism, FAO, the TCA cycle, and respiratory electron transport (*Figure 2*; Supplementary data online, *Figure S5*). Pathways associated with contraction generation were down-regulated, while those associated with mTOR signalling, collagen formation and insulin-like growth factor activity regulation were up-regulated. Empagliflozin treatment reversed these changes (*Figure 2*; Supplementary data online, *Figure S5* and *Table S3*). For additional details on significant pathway changes and their biological significance, see Supplementary data online, *Tables S2* and *S3*.

Collectively, these findings demonstrate that energy metabolism and hypertrophic signalling pathways are dysregulated early in R403Q hearts, as shown by transcriptomic data, and persist into advanced stages of HCM, as revealed by proteomic and phosphoproteomic analyses. Empagliflozin treatment normalizes these metabolic pathways and mitigates pathological hypertrophy, highlighting its potential to ameliorate the progression of metabolic and structural alterations in HCM.

### Empagliflozin normalizes substrate utilization and intracellular sodium in R403Q hearts

**Figure 3:**
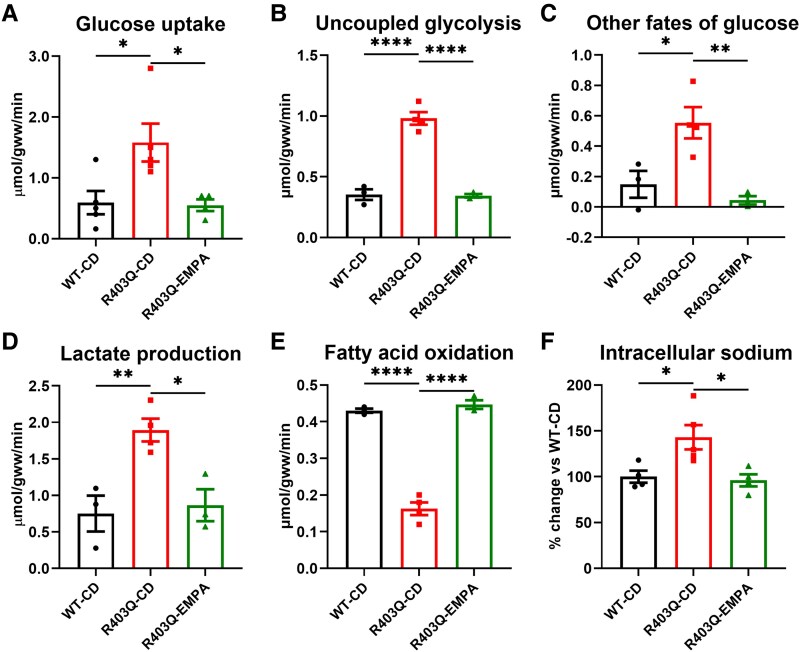
Empagliflozin decreases glucose uptake, improves glycolysis/glucose oxidation coupling and increases fatty acid oxidation and normalizes intracellular sodium levels in R403Q hearts. R403Q heart presented increased glucose uptake (*A*) associated with glycolysis/glucose oxidation uncoupling (*B*) with increase in ‘other fates of glucose’ (*C*) and increased lactate production (*D*). Furthermore, R403Q heart showed decreased fatty acid oxidation (*E*) and increased levels of intracellular sodium ([Na^+^]*_i_*) (*F*). A 16-week empagliflozin treatment switched cardiac metabolism back to fatty acid oxidation (*E*), decreased glucose uptake (*A*) and improved glycolysis/glucose oxidation coupling (*B*) as also evidenced by reducing ‘other fates of glucose’ (*C*), decreasing lactate production (*D*), and normalizing [Na^+^]*_i_* (*F*). Uncoupled glycolysis = glycolysis-glucose oxidation. Data shown are mean ± SEM. *n* = 3–4. *P*-values were obtained by one-way analysis of variance with Bonferroni multiple comparisons tests (adjusted *P*-value threshold <.05). *P < .05, **P < .01, and ****P < .0001

Building on these multi-omics findings, we used ^13^C NMR to directly measure substrate metabolic fluxes in R403Q hearts with and without EMPA treatment. R403Q hearts exhibited a metabolic shift from FAO to an inefficient glucose utilization. Specifically, FAO was reduced by 63% (*P* < .0001; *Figure 3E*) while glucose uptake increased by 167% (0.59 ± 0.19 μmol/gww/min in WT-CD vs 1.58 ± 0.31 μmol/gww/min in R403Q-CD; *P* < .05; *Figure 3A*). In absolute amounts, glucose uptake increased by 0.99 ± 0.37 μmol/gww/min and glucose oxidation increased only by 0.36 ± 0.06 μmol/gww/min (0.24 ± 0.04 μmol/gww/min in WT-CD vs 0.60 ± 0.05 μmol/gww/min in R403Q-CD; *P* < .01; Supplementary data online, *Figure S6*) resulting in increased uncoupling of glycolysis from glucose oxidation by 177% (0.35 ± 0.04 μmol/gww/min in WT-CD vs 0.98 ± 0.05 μmol/gww/min in R403Q-CD; *P* < .0001; *Figure 3B*). This uncoupling led to a 153% increase in lactate production (*P* < .01; *Figure 3D*) and 272% increase in other non-oxidative glucose pathways (*P* < .05; *Figure 3C*), resulting in inefficient glucose utilization as energy substrate. Empagliflozin treatment normalized these metabolic alterations in R403Q hearts by increasing FAO by 175%, restoring it as predominant cardiac energy substrate (*P* < .0001; *Figure 3E*). Additionally, glucose uptake decreased by 65% (*P* < .05; *Figure 3A*) and glycolysis/glucose oxidation coupling improved by 65% (*P* < .0001). This was further supported by a 54% (*P* < .05) reduction in lactate production and 92% (*P* < .01) decrease in other non-oxidative glucose pathways (*Figure 3B–D*). Additionally, normalization of acylcarnitine metabolism following EMPA treatment, as assessed by liquid chromatography–mass spectrometry (see Supplementary data online, *Figure S7*), further underscores its beneficial effect on dysregulated FAO. Moreover, intracellular sodium concentration ([Na^+^]*_i_*) which was elevated by 43% in R403Q hearts (*P* < .05), decreased by 33% with EMPA treatment (*P* < .05, *Figure 3F*). This suggests that the accumulation of glycolytic byproducts such as lactate and protons contributes to elevated [Na^+^]*_i_*, while improved glycolysis/glucose oxidation coupling reverses these abnormalities.

Together, these findings demonstrate a profound metabolic shift in R403Q hearts from FAO to inefficient glucose utilization, characterized by uncoupling of glycolysis from glucose oxidation and elevated intracellular sodium levels. Empagliflozin treatment effectively normalized these metabolic and sodium handling imbalances.

### Empagliflozin improves LV contractile function, cardiac energetics and efficiency in R403Q hearts

**Figure 4:**
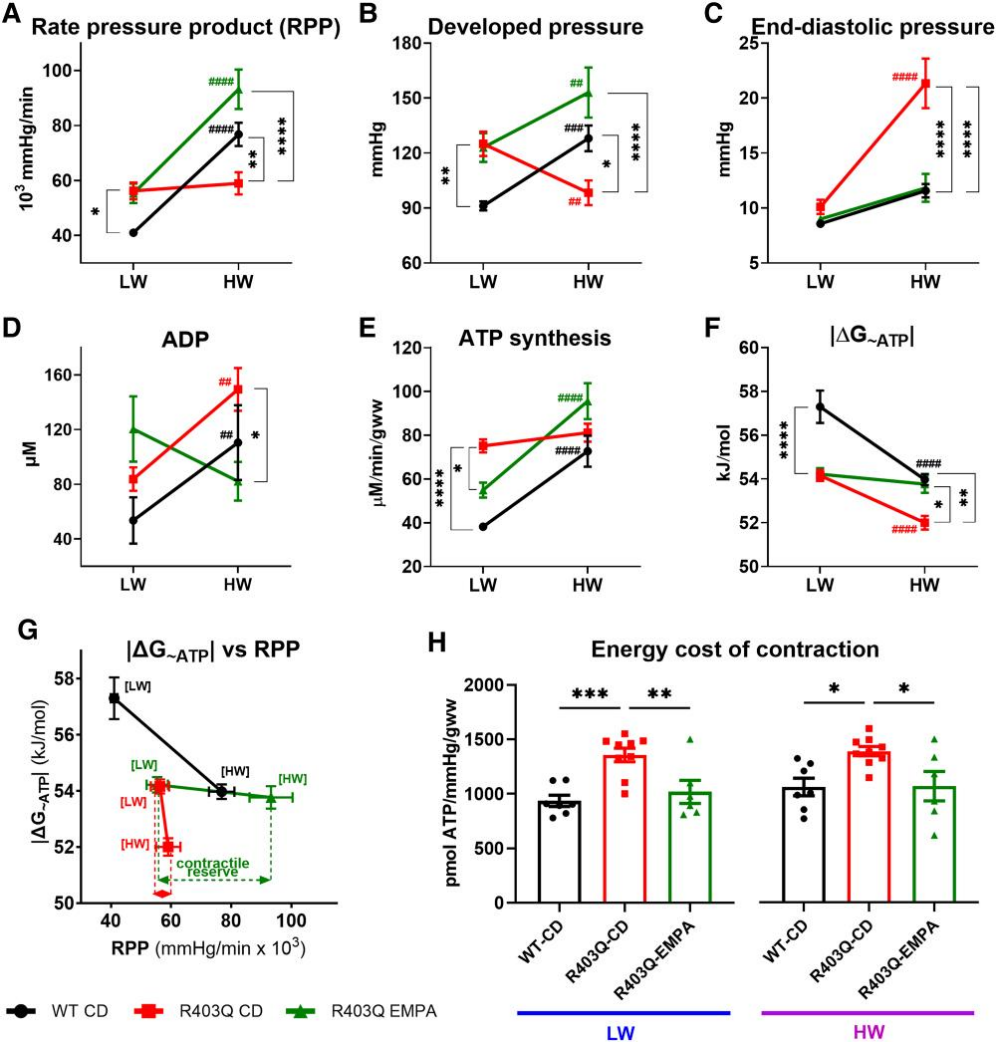
Empagliflozin improves cardiac contractile function, energetics, and efficiency in R403Q hearts. At low workload, R403Q hearts exhibited elevated rate pressure product (*A*) and developed pressure (*B*) and similar end-diastolic pressure (*C*) compared with control hearts. At high workload, while control hearts increased rate pressure product (*A*) and developed pressure (*B*) without significant changes in end-diastolic pressure (*C*), R403Q hearts failed to increase rate pressure product (*A*), displayed decreased developed pressure (*B*), and markedly increased end-diastolic pressure (*C*). After 16-weeks of empagliflozin treatment, R403Q contractile reserve improved as evidenced by increased rate pressure product (*A*) and developed pressure (*B*) at high workload, while maintaining stable end-diastolic pressure (*C*). R403Q hearts showed impaired cardiac energetics: R403Q hearts failed to adequately increase adenosine triphosphate synthesis (*E*) had elevated ADP levels (*D*) and showed a significant reduction in the free energy of adenosine triphosphate hydrolysis (|Δ*G*_∼ATP_|) (*F*), which was already lower than control at low workload. Empagliflozin treatment effectively increased adenosine triphosphate synthesis (*E*) decreased ADP levels (*D*), and stabilized |Δ*G*_∼ATP_| (*F*) under high workload conditions. The graph (*G*) demonstrates the interplay between cardiac energetics and contractile function, indicating that empagliflozin treatment enabled significant increases in rate pressure product at high workload with minimal impact on |Δ*G*_∼ATP_|. Additionally, empagliflozin treatment reduced the energy cost of contraction (*H*) at both low workload and high workload, indicating enhanced energetic efficiency. Data shown are mean ± SEM. *n* = 5–9. *P*-values were obtained by two-way repeated measures analysis of variance with Bonferroni multiple comparisons tests (adjusted *P*-value threshold <.05) to analyse the contractile function and ^31^P NMR data from the isolated beating heart, and one-way analysis of variance with Bonferroni multiple comparisons tests (adjusted *P*-value threshold <.05) to analyse energy cost of contraction. *P < .05, **P < .01, ***P < .001, and ****P < .0001 to denote significant differences between study groups; ^##^*P* < .01, ^###^*P* < .001, and ^####^*P* < .0001 to denote significant differences between high workload and low workload within a study group

R403Q hearts demonstrated impaired contractile reserve as evidenced by their inability to increase developed pressure (DevP) and rate pressure product (RPP) at high workload (HW), coupled with a significant increase in LV end-diastolic pressure (EDP) (*Figure 4A–C*).

At low workload (LW), R403Q hearts exhibited elevated DevP (by 37%, *P* < .01; *Figure 4A*) and RPP (by 37%, *P* < .05; *Figure 4B*) with a comparable EDP to control hearts (ns; *Figure 4C*). At HW, control hearts significantly increased DevP and RPP (40% and 87% vs LW, respectively; *P* < .0001 and *P* < .001; *Figure 4A* and *B*) without a significant rise in EDP (ns, *Figure 4C*). In contrast, R403Q hearts failed to increase RPP and showed a marked 111% rise in EDP compared with LW (*P* < .0001; *Figure 4C*).

Empagliflozin treatment markedly improved the contractile reserve in R403Q hearts. At HW, EMPA-treated R403Q hearts increased RPP by 68% (*P* < .0001; *Figure 4A*) and DevP by 24% (*P* < .01; *Figure 4B*) while maintaining stable EDP (ns; *Figure 4C*). Compared with untreated R403Q hearts, EMPA treated hearts at HW exhibited 56% higher DevP, 58% higher RPP (both *P* < .0001; *Figure 4A* and *B*), and 45% lower EDP (*P* < .0001; *Figure 4C*).

The reduced contractile reserve in R403Q hearts was associated with impaired cardiac energetics. Despite increased baseline ATP synthesis rate, the absolute value of free energy of ATP hydrolysis |Δ*G*_∼ATP_| was reduced, reflecting a mismatch between ATP supply and demand. At HW, ATP synthesis increased by 90% in control hearts (*P* < .0001) but failed to increase in R403Q hearts (ns; *Figure 4E*), further worsening |Δ*G*_∼ATP_|. Empagliflozin treatment did not affect |Δ*G*_∼ATP_| at LW but increased ATP synthesis by 74% at HW (*P* < .0001; *Figure 4E*) resulting in significantly increased |Δ*G*_∼ATP_| (*P* < .05; *Figure 4F*). Adenosine diphosphate (ADP) levels mirrored the |Δ*G*_∼ATP_| changes, with higher ADP in R403Q hearts unaffected by EMPA treatment at LW but significantly decreased at HW (45% reduction, *P* < .05; *Figure 4D*).

Graphical analysis of |Δ*G*_∼ATP_| and RPP underscores the intertwined relationship between cardiac energetics and contractile function (*Figure 4G*). Empagliflozin improved the contractile reserve in R403Q hearts, enabling a 68% increase in RPP at HW (*P* < .0001) with minimal impact on |Δ*G*_∼ATP_|. Additionally, EMPA reduced the energy cost of contraction by 25% at LW and 24% at HW (*P* < .01 and *P* < .05, respectively; *Figure 4H*), highlighting its efficacy in enhancing energetic efficiency in compromised R403Q hearts.

Together, these findings demonstrate impaired contractile function and energetics in R403Q hearts at LW, further deterioration at HW, and normalization following EMPA treatment. Empagliflozin enabled R403Q hearts to respond to increased workload by improving cardiac energetics and enhancing efficiency.

### Empagliflozin prevents cardiac hypertrophy and improves diastolic function in R403Q hearts

At 12 weeks of age, echocardiography of R403Q hearts revealed diastolic dysfunction without significant changes in total wall thickness compared with controls. Specifically, *E*/*A* ratio and *E*_m_ were reduced by 26% and 19%, respectively (*P* < .0001 and *P* < .001; Supplementary data online, *Figure S1*), indicating that diastolic dysfunction precedes LVH in this model, consistent with findings from human HCM.^36^ Baseline echocardiography at 9–10 weeks of age demonstrating similar pattern is shown in Supplementary data online, *Figure S8*.

**Figure 5:**
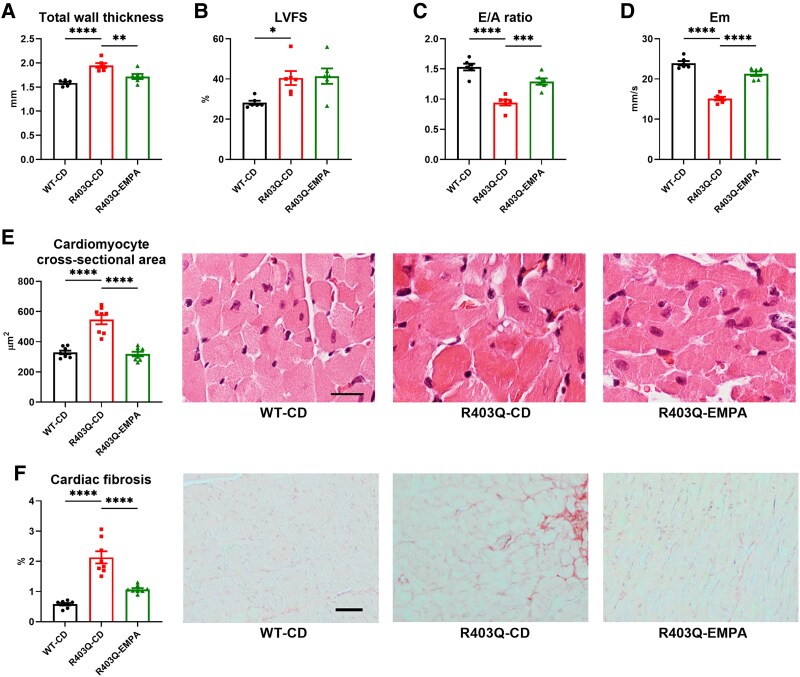
Empagliflozin prevents cardiac hypertrophy and improves diastolic function in R403Q hearts. R403Q hearts developed left ventricular hypertrophy as evidenced by increased total wall thickness (*A*) on echocardiogram and increased cardiomyocyte cross-section area (*E*) and fibrosis (*F*) in histopathology. Hypercontractility in R403Q hearts was evidenced by increased left ventricular fractional shortening (*B*). Furthermore, deteriorated left ventricular diastolic function was evidenced by reduced *E*/*A* ratio (*C*) and *E*_m_ (*D*). A 16-week empagliflozin treatment reduced cardiac hypertrophy and fibrosis (*A*, *E*, and *F*). Additionally, empagliflozin enhanced left ventricular diastolic function (*C* and *D*) without affecting hypercontractility (*B*). Data shown are mean ± SEM. *n* = 6 (for echocardiography) and 8 (for histopathology). *P*-values were obtained by one-way analysis of variance with Bonferroni multiple comparisons tests (adjusted *P*-value threshold <.05). *P < .05, **P < .01, ***P < .001, and ****P < .0001. Representative photomicrographs, hematoxylin–eosin for myocyte cross-section area analysis (*E*) or picrosirius red for cardiac fibrosis analysis (*F*), transmitted light, scale bar indicates 20 µm for myocyte cross-section area analysis (*E*) and 50 µm for cardiac fibrosis analysis (*F*). Additional histopathological images are shown in Supplementary data online, *Figure S9*

At the end of the study (age 25 weeks), R403Q hearts exhibited LVH as demonstrated by echocardiography and histopathology (see *Figure 5* and Supplementary data online, Figure  *S9*). Total wall thickness increased by 23% (*P* < .0001), while EMPA treatment decreased this by 12% (*P* < .01; *Figure 5A*). Histopathology showed a 66% increase in cardiomyocyte cross-sectional area and a 267% increase in cardiac fibrosis in R403Q hearts (both *P* < .0001); EMPA treatment reduced these by 42% and 50%, respectively (both *P* < .0001; *Figure 5E* and *F*). R403Q hearts again demonstrated LV diastolic dysfunction, with *E*/*A* ratio and *E*_m_ decreased by 39% and 37%, respectively (*P* < .0001 and *P* < .001; *Figure 5C* and *D*). Empagliflozin improved LV diastolic function, increasing the *E*/*A* ratio by 37% and *E*_m_ by 41% (both *P* < .0001; *Figure 5C* and *D*). As expected in an HCM model with hypercontractility, LV fractional shortening increased by 44% (*P* < .05; *Figure 5B*). Consistent with concentric remodelling, LV end-systolic and end-diastolic diameters were reduced by 25% (*P* < .05) and 11% (ns), respectively, with no significant changes following EMPA treatment (see Supplementary data online, *Figure S10*). These results were corroborated in adult isolated cardiomyocytes where chronic EMPA treatment improved the impaired calcium handling and sarcomere relaxation (see Supplementary data online, *Figure S15*). However, acute EMPA treatment in human induced pluripotent stem cell-derived cardiomyocytes (iPSC-CMs) harbouring the myosin R403Q mutation had no effect on these parameters (see Supplementary data online, *Figure S18*).

Strikingly, EMPA reversed LVH even when treatment was initiated at the fully developed HCM phenotype (see Supplementary data online, *Figure S11*). Moreover, we observed similar effects in a separate HCM model carrying the R92L mutation in cardiac troponin T,^37^ indicating that EMPA's benefits on cardiac hypertrophy and function in HCM are independent of the specific pathogenic sarcomere mutation (see Supplementary data online, *Figure S12*). Collectively, these findings show that EMPA has a potential to both *prevent* and *reverse* cardiac hypertrophy and diastolic dysfunction in HCM hearts.

#### Hypertrophic signalling

**Figure 6:**
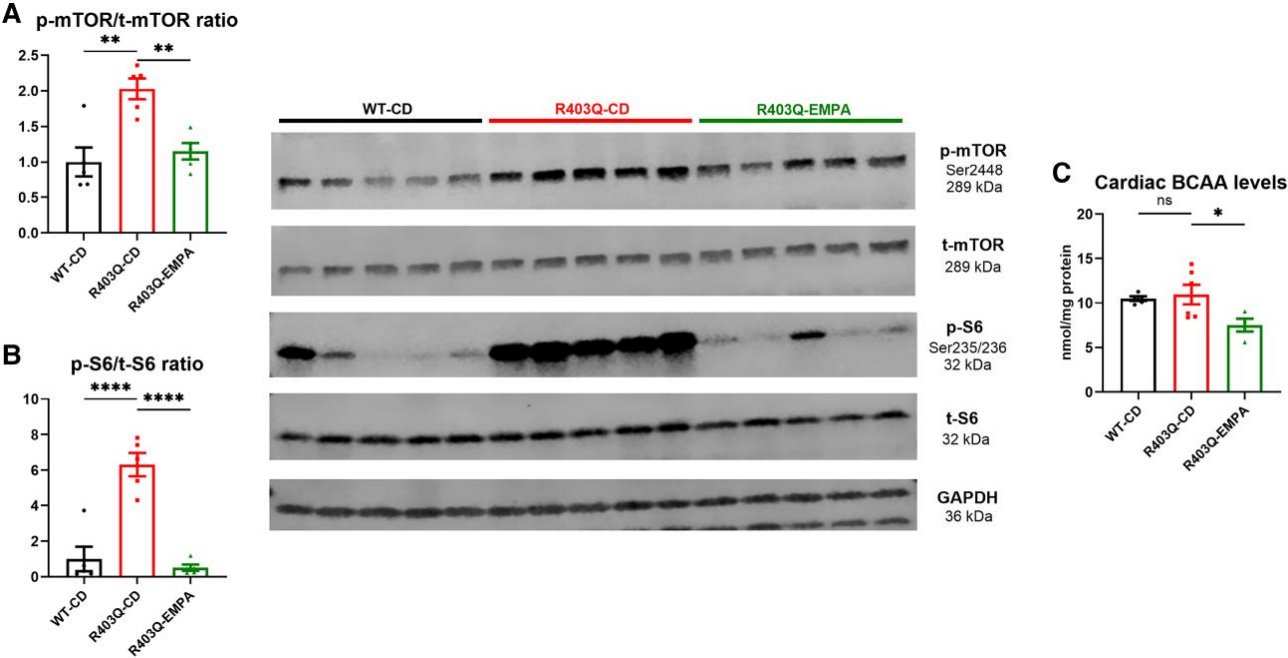
Empagliflozin attenuates mammalian target of rapamycin pathway activation and decreases cardiac branched-chain amino acids levels in R403Q hearts. Correspondingly to hypertrophy development, p-mTOR/t-mTOR ratio (*A*), a key regulator of growth, and p-S6/t-S6 ribosomal protein ratio (*B*), its major downstream effector, were increased in R403Q hearts as shown by western blot analysis. A 16-week empagliflozin treatment attenuated mammalian target of rapamycin pathway activation (*A* and *B*) and decreased cardiac branched-chain amino acids levels (*C*), a well-known mammalian target of rapamycin pathway activator. Data shown are mean ± SEM. The *n* = 5 (for western blot) and 4–6 (for cardiac branched-chain amino acid levels measurement). Uncropped western blot images are depicted in Supplementary data online, *Figure S13*. *P*-values were obtained by one-way analysis of variance with Bonferroni multiple comparisons tests (adjusted *P*-value threshold <.05). ns, not significant. *P < .05 and **P < .01, and ****P < .0001

The mTOR signalling pathway, a key regulator of growth and energy status, was significantly activated in R403Q hearts. Phospho-mTOR/total-mTOR and phospho-S6/total-S6 ribosomal protein ratios were increased by 103% (*P* < .01; *Figure 6A*) and a 532% (*P* < .0001; *Figure 6B*), respectively, while EMPA treatment reduced these by 43% (*P* < .01) and 92% (*P* < .0001; *Figure 6A* and *B*; uncropped images are shown in Supplementary data online, *Figure S13*). Transcriptomics and proteomics at 12 and 25 weeks of age, respectively, also indicated an up-regulation of the mTOR signalling in R403Q hearts and its down-regulation by EMPA treatment (see Supplementary data online, *Tables S1* and *S3*). Interestingly, myocardial BCAA concentrations, a major stimulus for mTOR activation,^38,39^ trended 4% higher in R403Q hearts (ns) and were reduced by 31% with EMPA treatment (*P* < .01; *Figure 6C*), consistent with transcriptomic findings of increased BCAA catabolism with EMPA (*Table 1*). Liquid chromatography–mass spectrometry corroborated these findings, showing elevated levels of individual BCAAs: isoleucine, leucine and valine in R403Q hearts, normalized with EMPA treatment (see Supplementary data online, *Figure S14*). Interestingly, a 16-week treatment with 3,6-dichlorobenzo[*b*]thiophene-2-carboxylic acid (BT2), which activates BCAA oxidation,^40^ was associated with attenuation of LVH in R403Q hearts (see Supplementary data online, *Figure S17*). Taken together, these findings suggest the critical role of impaired BCAA catabolism and mTOR signalling in R403Q mutation-induced HCM, which is effectively mitigated by EMPA treatment, even after as short as 2 weeks of administration. Of note, trends towards improved plasma BCAA levels, blood glucose, ketone, and lactate homeostasis were observed after 16 weeks of EMPA treatment (see Supplementary data online, *Figure S16*).

## Discussion

This study demonstrates the therapeutic potential of SGLT2i EMPA in a murine model of HCM induced by the R403Q cardiac myosin mutation. Empagliflozin improved coupling between glycolysis and glucose oxidation, normalized elevated intracellular sodium, and enhanced mitochondrial function in HCM hearts. These metabolic improvements, likely driven by the restoration of mitochondrial oxidative capacity, were associated with reduced LVH, ameliorated fibrosis, improved LV diastolic function and contractile reserve.

Our study identifies several interrelated mechanisms of HCM pathophysiology that may be improved by treatment with SGLT2i.

### Impaired myocardial energetics

Hypertrophic cardiomyopathy mutations in sarcomere-associated genes lead to inefficient power generation by the sarcomere, resulting in up to five-fold increased ATP consumption by myosin ATPase.^3,41^ These mutations also impair the transition of myosin heads to the super-relaxed state,^42^ increasing ATP hydrolysis contributing to diastolic dysfunction, ATP wastage, and reduced efficiency of power generation. As a result, HCM hearts exhibit an energetic deficit, characterized by reduced PCr/ATP ratio and a decreased free energy of ATP hydrolysis |Δ*G*_∼ATP_|.^4,26,43^ Our findings corroborate this, revealing a significant energetic deficit and increased energy cost of contraction in R403Q hearts.

The mismatch between ATP supply and demand in HCM elevates ADP levels and decreases |Δ*G*_∼ATP_|. When |Δ*G*_∼ATP_| declines to the critical threshold of 52–53 kJ/mol, it approaches the minimal energy required for sarcoplasmic/endoplasmic reticulum Ca^2+^-ATPase (SERCA) function.^44^ Further reductions in ATP flux impair diastolic calcium reuptake, leading to diminished myocardial relaxation,^44,45^ elevated LVEDP and reduced contractile reserve—the heart's capacity to enhance contractility under increased workload^28,29,45–47^ Elevated ADP has been shown to contribute to impaired myocardial relaxation in HCM,^4^ consistent with our data showing diastolic dysfunction and decreased contractile reserve in R403Q hearts. When SERCA's function declines, other Ca^2+^ extruding mechanisms are up-regulated and lead to further cytosolic [Na^+^]*_i_* accumulation^48^ a phenomenon observed in HCM.^16,49^ Recently, EMPA was shown to improve mitochondrial respiratory function and cardiomyocyte calcium handling by modulation of SERCA in a murine heart failure with preserved ejection fraction (HFpEF) model.^50^ In our study, EMPA improved mitochondrial ATP synthesis, as measured by ^31^P NMR magnetization transfer, consistent with previous studies demonstrating the beneficial effects of SGLT2i on cardiac energetics in both diabetic^9,24,51,52^ and non-diabetic cardiomyopathy models.^53–55^ Notably, the causal relationship between cardiac energetics and function in HCM is further supported by our previous study, where saturating mitochondria with butyrate—an accessible energy substrate for mitochondrial ATP synthesis—acutely improved energetics and function of isolated beating R403Q hearts.^56^

### Uncoupled glycolysis

During foetal development, cardiomyocyte proliferation and maturation occur under low oxygen conditions, primarily relying on anaerobic glycolysis for both energy production and nucleotide synthesis. After birth, increased oxygen availability promotes a transition to adult mitochondria capable of efficient ATP synthesis through oxidative phosphorylation. This shift is accompanied by suppression of mTOR signalling, facilitating a metabolic transition from glycolysis to glucose oxidation and FAO as the primary energy sources.^9,14,57^

In HCM, the heart reverts to a foetal metabolic profile despite adequate oxygen and nutrients.^58,59^ This reversal is characterized by mTOR signalling activation, a preferential reliance on glycolysis, and a reduced oxidative phosphorylation. Resulting uncoupled glycolysis may lead to the accumulation of metabolic byproducts such as lactate and free H^+^ contributing to elevated [Na^+^]*_i_*.^14^ Notably, EMPA has been shown to reduce blood lactate levels in a murine HFpEF model.^50^ Accumulation of glucose-6-phosphate may further activate mTOR signalling, and induce cellular stress, contractile dysfunction, and cardiac remodelling through pathways such as the pentose phosphate pathway, hexosamine biosynthetic pathway,^60,61^ and the *o*-GlyNAcylation^62^ of proteins. In this study, we assessed the collective flux through these pathways as ‘other fates of glucose’. Indeed, they were elevated in HCM and normalized by EMPA treatment (*Figure 3C*).

A plausible mechanism for the treatment effect is that increased TCA cycle flux induced by EMPA enhances glucose oxidation,^63^ reducing excess glucose available for biosynthetic pathways and lactate production. This improved coupling may alleviate metabolic byproduct accumulation and associated cellular stress, contributing to improved metabolic efficiency. These effects may extend beyond the heart, as we have previously demonstrated that EMPA improved insulin sensitivity in R403Q mice.^64^ Insulin, in turn, directly stimulates mitochondrial glucose oxidation, enabling mitochondria to manage increased glucose uptake with reduced lactate production.^65^

Thus, our findings align with prior studies showing that EMPA increases mitochondrial capacity for glucose oxidation.^54,57,63^ In our study, this effect was associated with lower lactate production, normalization of elevated [Na^+^]*_i_*, improved calcium handling (see Supplementary data online, *Figure S15*) and enhanced diastolic function. Collectively, these observations underscore EMPA's potential to counteract adverse metabolic remodelling in HCM, offering a promising therapeutic avenue for mitigating disease progression.

### Role of [Na^+^]*_i_* in hypertrophic cardiomyopathy and potential relationship to arrhythmia prevention

Our results confirm previous findings of elevated intracellular sodium [Na^+^]*_i_*^15,16,49^ and mitochondrial dysfunction in HCM.^7,8,66^ This aligns with the reciprocal relationship between elevated [Na^+^]*_i_* and decreased mitochondrial calcium ([Ca^2+^]_MITO_), which impairs ATP synthesis and increases reactive oxygen species (ROS) production.^19,51^ The resulting ROS accumulation further exacerbates the energetic deficit and disrupts ion homeostasis.^67^ Notably, inhibition of ROS has shown therapeutic promise in HCM animal studies.^2^

Elevated [Na⁺]*_i_* has additional consequences, including reduced extrusion of calcium and protons via the plasma membrane Na⁺/Ca^2^⁺ and Na⁺/H⁺ exchangers leading to slowed relaxation and cytosolic acidification.^16,49,68^ Consistent with the literature,^3,69^ our study observed impaired calcium handling associated with increased sarcomere shortening and reduced relaxation in R403Q cardiomyocytes, which improved after EMPA treatment (see Supplementary data online, *Figure S15*).

Given that elevated [Na⁺]*_i_*–related changes in ion homeostasis contribute not only to diastolic dysfunction but also to compromised electrophysiology,^70^ it is notable that SGLT2i have demonstrated anti-arrhythmic effects in several clinical scenarios.^71^ Together with our data, this suggests the potential for EMPA to reduce ventricular arrhythmias and sudden cardiac death (SCD) in HCM, though this was not directly assessed in our study.

### Relationship between metabolic reprogramming and elevated [Na^+^]*_i_*

A switch of energy substrate preference away from FAO is known to occur in pathological hypertrophy and HF.^72^ A similar metabolic substrate switch has been suggested in HCM by metabolomic studies,^66^ but never confirmed by measurement of substrate oxidation fluxes. Our data provide the first evidence that EMPA can restore FAO and correct the uncoupled glycolysis observed in HCM, thereby normalizing substrate oxidation fluxes.

While a direct effect of SGLT2i on sodium transport has been suspected,^73,74^ elevated myocardial [Na^+^]*_i_* in HCM may also result from increased uncoupled glycolysis. Cytosolic acidification, driven by glycolysis, activates the sarcolemmal Na^+^/H^+^ exchanger leading to sodium overload.^13,14,75^ In this context, our observation that EMPA decreases myocardial [Na^+^]*_i_* in R403Q model may stem from its ability to improve coupling between glycolysis and glucose oxidation.

Notably, increased [Na^+^]*_i_* exacerbates energetic inefficiency by elevating ATP demand via Na^+^/K ^+^ -ATPase^49^ and reducing mitochondrial calcium [Ca^2+^]_MITO_,^19,51,76^ thereby impairing ATP production and further diminishing contractile efficiency.^14,75^ The reversal to foetal gene expression,^54,57^ reduction in fatty acid transport,^53,77^ increase in [Na^+^]*_i_*^22^ and enhanced mTOR signalling^78,79^ likely contribute to the decrease in FAO observed in R403Q hearts—all of which are improved by EMPA treatment.

### Branched-chain amino acid catabolism

Dysfunction in BCAA metabolism has been implicated in cardiac hypertrophy and failure, with suppression of BCAA catabolic pathways observed in both murine and human HF.^80^ Supressed cardiac BCAA catabolism inhibits PDH^81^ over-activates mTOR,^82^ and promotes glycolysis/glucose uncoupling and cardiac hypertrophy, respectively.^38,39,83^ Elevated BCAA levels are associated with adverse outcomes in HF patients,^84,85^ and contribute to cardiac injury.^80,86,87^

In contrast, facilitating BCAA catabolism, has shown cardioprotective effects in HF.^40,80^ Pharmacological activation of BCAA catabolism attenuates HF in mice subjected to transverse aortic banding, ischaemia/reperfusion, and myocardial infarction.^80–82^ Plasma BCAA levels and other metabolic parameters in our mice are shown in Supplementary data online, *Figure S16*.

Our findings indicate that EMPA reduces myocardial BCAA levels by enhancing BCAA catabolism, suppressing mTOR activation, and improving remodelling, energetics, and function in HCM. Furthermore, treatment with 3,6-dichlorobenzo[*b*]thiophene-2-carboxylic acid (BT2), a potent inhibitor of branched-chain ketoacid dehydrogenase kinase that activates BCAA oxidation,^40^ resulted in decreased cardiac hypertrophy in mice with R403Q mutation (see Supplementary data online, *Figure S17*).

These observations are consistent with prior studies demonstrating that improved BCAA catabolism enhances cardiac energetics and function in HF models, as well as in patients treated with EMPA.^54,88,89^ A recent mechanistic study further supports this, showing that EMPA increases coenzyme A production in the heart, thereby supporting fuel usage, including BCAAs and lactate.^63^ Thus, our findings align with broader research linking enhanced BCAA catabolism to improved cardiac function and provide a potential mechanistic link between enhanced mitochondrial oxidation and attenuation of LVH in HCM.

### Other potential mechanisms of empagliflozin's benefit

One proposed mechanism of EMPA's benefit is a direct effect on myosin function.^90^ This hypothesis has been suggested by studies identifying SGLT2i as modulators of sarcomere relaxation via changes in myofilament properties.^91,92^ However, our data do not strongly support this hypothesis. Acute EMPA treatment in human iPSC-CMs harbouring the myosin R403Q mutation did not alter calcium handling or relaxation (see Supplementary data online, *Figure S18*), despite the beneficial effect of chronic treatment in adult isolated cardiomyocytes (see Supplementary data online, *Figure S15*). If EMPA was acting directly on myosin, its effects should have been evident in both mature cardiomyocytes and iPSC-CMs, given that the myosin mutation remains constant regardless of cardiomyocyte type. Additionally, even chronic EMPA treatment failed to normalize the diastolic sarcomere length in isolated adult R403Q cardiomyocytes (see Supplementary data online, *Figure S15*), further arguing against a direct effect on myosin or the sarcomere.

A second potential mechanism involves EMPA's influence on sodium handling.^69^ Elevated intracellular sodium ([Na⁺]*_i_*) is a hallmark of HCM and contributes to impaired calcium handling and diastolic dysfunction. While the Na⁺/Ca^2^⁺ exchanger has been identified as pivotal in the SGLT2i-mediated mechanism underlying enhanced relaxation in HCM,^69^ the lack of effect in iPSC-CMs may be due to their lower sodium channel density or immaturity, which could obscure EMPA's benefits on sodium transport. Thus, the lack of an effect in iPSC-CMs does not necessarily negate EMPA's potential impact on sodium handling in HCM, and the significant improvements in mature cardiomyocytes suggest that sodium handling remains a plausible target for EMPA in HCM.

Another possible mechanism involves EMPA's metabolic effects via an aestivation-like hypometabolic state. This hypothesis posits that SGLT2i induce a systemic metabolic shift that mimics energy conservation during prolonged periods of starvation. This shift includes skeletal muscle serving as an energy and nitrogen reservoir, the liver producing urea and ketones, and the heart utilizing fatty acids, ketones, and BCAAs as fuel sources.^93^ Supporting this, our previous work has shown an increase in the glucagon/insulin ratio after EMPA treatment, consistent with the metabolic profile of aestivation.^64^

A more recent mechanistic explanation involves the direct activation of the enzyme pantothenate kinase 1 (PANK1) by EMPA. Pantothenate kinase 1 is the rate-limiting enzyme in the conversion of pantothenate (vitamin B5) to coenzyme A (CoA), a critical cofactor for all major metabolic pathways. Activation of PANK1 by EMPA increases CoA production, which enhances mitochondrial oxidative capacity and supports flux through the PDH complex, FAO, and BCAA oxidation.^63^

Regardless of the exact molecular target, the unifying mechanism underlying EMPA's benefits appears to be the enhancement of mitochondrial oxidative metabolism. By increasing mitochondrial ‘furnace’ capacity, EMPA reduces accumulation of metabolic byproducts including protons and lactate, reduces glucose diversion into biosynthetic pathways, and suppresses mTOR activation through enhanced BCAA catabolism. These metabolic adaptations likely contribute to the observed improvements in energetics, diastolic function, and structural remodelling in HCM.

### Limitations

First, while SGLT2i have shown anti-arrhythmic properties,^71^ the potential impact of EMPA on ventricular arrhythmias and SCD in HCM was not evaluated in this study. Future investigations are needed to explore this aspect. Our study focused primarily on EMPA's effects on cardiac metabolism, function, and phenotype in HCM, as HF remains the predominant cause of disease-related morbidity and mortality in HCM.^94,95^ Second, we limited our study to male R403Q mice due to the more consistent manifestation of the HCM phenotype in males.^25,96^ While this approach models aspects of human HCM, sex-based differences in disease progression and treatment response may warrant further investigation in female models. Importantly, our findings from the troponin R92L HCM model, which exhibits the phenotype in both sexes, indicate that EMPA provides improvement in both male and female mice. Third, while mTOR signalling is known to play a key role in HCM pathophysiology, and inhibition of this pathway (e.g. with rapamycin) has been shown to ameliorate the HCM phenotype in various models,^97,98^ proving a causal role for mTOR in EMPA's effects is challenging. The degree of cardiac mTOR activation in HCM is potentially dependent on several factors, including genetic predispositions associated with different HCM pathogenic mutations, HCM phenotype and severity, and coexisting conditions linked to mTOR activation such as Type 2 diabetes mellitus or arterial hypertension.^99,100^ The influence of these factors on the therapeutic effects of EMPA in HCM warrants future research. Currently, there is no viable mTOR knockout model available to conclusively establish this pathway's involvement.^99^ Further studies employing advanced genetic models or targeted mTOR inhibition may help elucidate the precise role of mTOR in EMPA's therapeutic effects on HCM. Fourth, potential confounding variables should be considered, as they may influence the observed outcomes in clinical scenarios involving HCM patients. These include genetic variations, differences in metabolic states (e.g. concurrent Type 2 diabetes), or the effects of concomitant medications, such as beta-blockers known to impact glucose and lipid metabolism.

### Clinical relevance

Recent advancements, such as the introduction of cardiac myosin inhibitors, have provided effective treatment options for obstructive HCM. However, non-obstructive HCM remains without targeted therapies, leaving a significant unmet clinical need. This is particularly critical as HF has emerged as the predominant cause of disease-related morbidity and mortality in HCM patients, highlighting an urgent need for innovative treatments.^94,95^

Our findings suggest that SGLT2i, such as EMPA, could address this gap by offering a novel therapeutic option for non-obstructive HCM. The observed amelioration of LVH and improved diastolic function with EMPA indicate its potential to alter the natural history of the disease. By preventing or attenuating the progression of hypertrophy, SGLT2i could provide early intervention opportunities for genotype-positive patients, even before the onset of symptoms or the development of the HCM phenotype.

Given its remarkable ability to reverse hypertrophy in preclinical models, EMPA warrants further investigation for its potential synergistic effect with myosin inhibitors in obstructive HCM and, potentially, non-obstructive HCM if these therapies are extended to broader patient populations. Furthermore, EMPA's favourable safety profile, cost-effectiveness, and demonstrated efficacy in other cardiovascular conditions make it an attractive candidate for clinical investigation and treatment across all HCM subtypes. Its capacity to address both metabolic and structural remodelling highlights its potential to slow disease progression, improve quality of life, and reduce the risk of HF in this underserved population.

This work aligns with the growing body of evidence supporting the utility of SGLT2i in cardiovascular diseases and highlights their promise as a potential therapeutic approach for HCM, offering hope for early and effective intervention strategies.

## Conclusions

This study provides robust evidence supporting the therapeutic potential of the SGLT2i EMPA in ameliorating several key aspects of HCM pathology induced by the R403Q mutation in cardiac myosin. Our findings underscore the central role of metabolic dysregulation in HCM and point to the potential of SGLT2i as valuable therapeutic agents. These findings advocate for further clinical evaluation of EMPA and potentially other SGLT2i in HCM, to explore their full clinical utility and to elucidate the underlying mechanisms contributing to their beneficial effects. This could pave the way for more targeted therapies that address the complex metabolic and structural changes observed in HCM, opening avenues for improved management of this challenging condition.

## Supplementary Material

ehaf324_Supplementary_Data

## Declarations

### Data Availability

Data supporting the findings of this study are available from the corresponding author upon reasonable request.

### Pre-registered Clinical Trial Number

None supplied.
