# Supplementary material for: Empagliflozin enhances metabolic efficiency and improves left ventricular hypertrophy in a hypertrophic cardiomyopathy mouse model
Source: Eur Heart J. 2025 May 21;46(40):4105–19. doi: 10.1093/eurheartj/ehaf324 (PMC12539926; doi:10.1093/eurheartj/ehaf324)
Supplement: ehaf324_Supplementary_Data [file ehaf324_supplementary_data.pdf]

# The SGLT2 Inhibitor Empagliflozin Enhances Metabolic Efficiency and Ameliorates Left Ventricular Hypertrophy in Myosin R403Q Mutant Hypertrophic Cardiomyopathy Mice

Tomas Baka, Jarrod Moore, Fuzhong Qin, Salva R Yurista, Aifeng Zhang, Huamei He, Jordan M Chambers, Dominique Croteau, Raghuveera K Goel, Hunter Smith, Miranda C Wang, Christopher S Chen, Ion A Hobai, Martina Rombaldova, Ondrej Kuda, Jil C Tardiff, James A Balschi, David R Pimentel, Christine E Seidman, Jonathan G Seidman, Andrew Emili, Wilson S Colucci, Ivan Luptak

## Supplementary material

**Supplementary Methods** pages 2-15

**Supplementary Figures** pages 16-33

**Supplementary Tables** pages 34-41

## Supplementary Methods

### Supplementary animal studies

To determine the effect of EMPA on developed HCM, a 16-week EMPA treatment was initiated in mice aged 24 weeks and the cardiac phenotype (LV hypertrophy) was assessed longitudinally. Adult male mice (20-30 weeks old) harboring cardiac myosin R403Q mutation were shown to present hallmarks of developed hypertrophic cardiomyopathy (HCM), including LV hypertrophy, cardiac fibrosis and cardiomyocyte disarray.[1–3] At the end, the effect of EMPA on cardiomyocyte contractility and $Ca^{2+}$ handling was measured in isolated myocyte studies.

To increase generalizability of our findings with respect to other HCM pathogenic sarcomere mutations, a 16-week EMPA treatment was initiated in 9-10-week-old mice harboring R92L mutation in cardiac troponin T; the cardiac function and phenotype were determined by echocardiography and histopathology at the end of the experiment. R92L mutant hearts were shown to develop significant hypertrophic phenotype and energetic deficit.[4,5] These mice were developed and kindly provided by Dr. Jil Tardiff (University of Arizona, Tucson, USA).[6]

To determine the role of BCAA metabolism in cardiac hypertrophy, 9-10-week-old R403Q mice were started a on control diet or diet enriched with 3,6-dichlorobenzo[b]thiophene-2-carboxylic acid (BT2), a potent inhibitor of branched chain ketoacid dehydrogenase kinase (BCKDK), at 450 mg BT2 per 1 kg diet. BT2 administration leads to the activation of BCAA oxidation, reduced plasma BCAA and branched-chain α-ketoacids (BCKA) levels and cardioprotective effects after a wide range of cardiac insults, including pressure overload, myocardial infarction, and ischemia-reperfusion.[7] After 16 weeks of treatment, BT2's effect on cardiac function and phenotype was assessed by echocardiography.

## Echocardiography

A VisualSonics Vevo 2100 high-resolution imaging system equipped with a 22- to 55-mHz MS550D transducer and Vevo offline analytic software (Toronto, Canada) were used for data capture and analysis. Mice were anesthetized with isoflurane at a concentration of 2.5% for anaesthesia induction and then 1.5% for anaesthesia maintenance. To assess LV dimensions and systolic function, the heart was imaged in the 2-D parasternal short-axis view, and an M-mode echocardiogram of the mid-ventricle was recorded at the level of papillary muscles. LV anterior (AWT) and posterior wall thickness (PWT), LV end-diastolic (EDD) and end-systolic (ESD) dimensions were measured, and total wall thickness and LV fractional shortening (FS = (EDD–ESD)/EDD × 100) were calculated. To assess LV diastolic function, pulsed-wave Doppler images were collected in the apical four-chamber view to record the mitral Doppler flow spectra and measure peak early (E) and late (A) mitral inflow velocities and calculate E/A ratio; tissue Doppler images were collected in the parasternal short-axis view to measure myocardial peak early diastolic velocity (Em).[8,9]

## Determination of left ventricular contractile function and high-energy phosphates in isolated beating hearts by $^{31}$P NMR spectroscopy

Left ventricular (LV) contractile function and high-energy phosphates were measured simultaneously in isolated retrograde-perfused Langendorff heart. Briefly, hearts were perfused with Krebs-Henseleit buffer containing 5.5 mM glucose and 0.4 mM mixed long chain fatty acids (bound to 1% albumin) of 60% palmitic acid, 8% palmitoleic acid, 24% oleic acid, and 8% linoleic acid; and 50 µU/ml insulin. Water-filled balloon was inserted into the LV. After stabilization, balloon volume was adjusted to achieve an LV end-diastolic pressure (EDP) of 8 to 9 mmHg and held constant during the protocol. The LV developed pressure (DevP) was calculated as: DevP = systolic pressure – end-diastolic pressure (LVEDP). LV workload was changed by increasing the concentration of $CaCl_2$ in the Krebs-Henseleit buffer from 2 to 4

mmol/l and increasing the pacing rate from 450 to 600 bpm. Rate pressure product (RPP = DevP × heart rate) was calculated to estimate the work performed. Simultaneously, [ATP], [phosphocreatine (PCr)], [inorganic phosphate (Pi)], and intracellular pH (pH) were measured by $^{31}P$ NMR using a Varian spectrometer at 161.4 MHz (Varian, Palo Alto, CA, USA). Cytosolic creatine concentration ([Cr]) was determined from freeze-clamped hearts using HPLC and used for ADP and $\Delta G_{\sim ATP}$ calculations.[10,11]

The cytosolic creatine concentration ([Cr]) was calculated as the difference between total creatine concentration [$Cr_{total}$] measured by High Performance Liquid Chromatography (HPLC)[12] and phosphocreatine ([PCr]) measured by $^{31}P$ NMR. The free [ADP] was calculated using the creatine kinase reaction (Equation 1) assumed to be at equilibrium, where $K_{eq}$ = 1.66 × $10^9$ $M^{-1}$.[13–15]

Equation 1: $[ADP] = ([ATP][Cr])/([PCr][H^+])K_{eq}$

The free energy of ATP hydrolysis ($\Delta G_{\sim ATP}$) was calculated by using the Equation 2, where $\Delta G_0$ (−30.5 kJ/mol) is the value of $\Delta G_{\sim ATP}$ under standard conditions, R = 8.314 J/mol·K, and T = 310 K. The value of $\Delta G_{\sim ATP}$ is negative, which denotes that the reaction is exergonic or energy-releasing. For the sake of clarity, we use the absolute value of $\Delta G_{\sim ATP}$, i.e. $|\Delta G_{\sim ATP}|$.[13–15]

Equation 2: $|\Delta G_{\sim ATP}| = |\Delta G_{0\sim ATP} + RT\ ln[ADP][Pi]/[ATP]|$

ATP synthesis rates were measured with the 2-site saturation transfer technique by applying a low-power narrow-band radiofrequency in order to saturate γ-ATP resonance and measure changes in the PCr and Pi resonance.[13–15] ATP synthesis rates were measured with the 2-site saturation transfer technique. A low-power narrow-band radiofrequency was applied in order to saturate γ-ATP resonance and measure changes in the PCr and Pi resonance. Spectra were acquired with 4.8 s ($M_\infty$) a selective saturating pulse and without ($M_0$). To eliminate direct attenuation of the observed resonance by the γ-ATP targeted saturation pulse, a same-power radiofrequency pulse was used and targeted at an equal frequency offset

downfield from the observed resonance during the $M_0$ acquisition. This allowed control for "radiofrequency spillover" by a symmetrical irradiation targeted at the same offset on the opposite side of the Pi resonance. The unidirectional pseudo–first-order rate constant of ATP synthesis was calculated using the formulae: kf = $(M_0/M_\infty)/T1\ M_\infty$ and flux = kf [Pi], where T1 is the intrinsic longitudinal relaxation time for Pi, [Pi] and $M_0$ and $M_\infty$ are magnetizations of Pi at 0 ($M_0$) and 4.8 s ($M_\infty$).

The energy cost of contraction was calculated as the amount of ATP synthesized to perform work (cost of contraction = ATP synthesis/RPP).[14,15]

**Determination of $^{13}C$ substrate oxidation and $[Na^+]_i$ in isolated beating hearts**

To determine substrate oxidation rates, hearts were perfused with $^{13}C$-enriched fatty acids (U-$^{13}C$ labeled fatty acid mix, Cambridge Isotope, CLM-8455, 0.4 mM) and 1-$^{13}C$ labeled glucose (Cambridge Isotope, CLM-420, 5.5 mM).[16] Proton-decoupled $^{13}C$ NMR (102.8 MHz, 9.4T) spectra of cardiac tissue were acquired. The contributions of each substrate to the oxidative metabolism and the rate of anaplerosis were determined using the $^{13}C$ isotopomers peak areas of the C3 and C4 of glutamate by modeling the tricarboxylic acid cycle fluxes as previously described.[17–19] Glucose uptake was measured by perfusion with 2-deoxyglucose (2-DG) and subsequent accumulation of 2-DG-phosphate measured with $^{31}P$ NMR.[20] $^{13}C$-labelled lactate was measured in the perfusate to determine the fate of pyruvate.

Intracellular sodium levels $[Na^+]_i$ in isolated hearts were determined by $^{23}Na$ MRS as described previously.[11] Briefly, hearts were perfused with Krebs-Henseleit buffer containing glucose (10 mmol/l), pyruvate (0.5 mmol/l), NaCl (118 mmol/l), KCl (5.9 mmol/l), $MgSO_4$ (1.2 mmol/l), $NaHCO_3$ (25 mmol/l), $CaCl_2$ (3.4 mmol/l), and a sift reagent thulium(III) 1,4,7, 10-tetraazacyclododecane-N,N',N",N'"-tetra(methylene-phosphonate ($TmDOTP5^-$ at 3.5 mmol/l). A balloon filled with water (volume adjusted to achieve an LVEDP of 8–9 mmHg) was inserted into the LV. A Varian spectrometer at 105.5 MHz was used to acquire the $^{23}Na$ NMR spectra.

For quantification, an average of five subsequent spectra was used. Quantification was performed relative to a reference resonance of a capillary placed next to the heart filled with 20 mmol/l TmDOTP5$^-$ and 12 µL of 736 mmol/l $Na^+$. To determine the heart dry weights at the end of the protocol, hearts were dried at 60°C for 48h. $[Na^+]_i$ was calculated using the formula: $[Na^+]_i$ = (NMR area)/(NMR standard area) × ($Na^+$ standard content)/$V_i$], where $V_i$ is intracellular volume determined from dry weight and $^{31}$P MRS. [11,21,22]

**Heart weight, histology, western blot analysis and BCAA levels measurement**

Mice were euthanized, hearts were harvested and weighed at the end of the study. LV samples were fixed in fixed in 10% buffered formalin, embedded in paraffin, sectioned, and stained. Myocyte cross-sectional area, and fibrosis (Picrosirius Red) were measured and analyzed, as previously described.[8,9] To assess myocyte cross-sectional area, sections were stained with hematoxylin and eosin and examined under a light microscope. Five random fields from two sections per animal were analyzed and 50 myocytes per animal were quantified. The cardiomyocyte cross-sectional area was calculated using ImageJ software (National Institutes of Health, Bethesda, MD, USA). To assess fibrosis, sections were stained with Picrosirius Red Stain Kit (Polysciences, Warrington, PA, USA) and examined under a light microscope. Mean percentage of fibrosis was determined from 10 random fields per heart and quantified using ImageJ software.

For western blot analysis, frozen heart samples were homogenized and sonicated in ice-cold lysis buffer.[23] Isolated protein concentration was measured by Pierce BCA assay kit (Catalog No. 23225, Thermo Fisher Scientific, Waltham, MA, USA). Thereafter, the SDS-PAGE on 4–20% gradient gels (Bio-Rad, Hercules, CA, USA) was followed by protein transfer to a PVDF transfer membrane with fluorescent capability (MilliporeSigma, St. Louis, MO, USA). Then, blots were blocked with the Intercept Blocking Buffer (Li-Cor, Lincoln, NE, USA) incubated with primary and appropriate secondary antibody (Li-Cor). Signal was detected

using the Li-Cor Odyssey scanner. The primary antibodies were used at the following dilutions: total-mTOR (Catalog No. 2972, Cell Signaling, Danvers, MA, USA) at 1:500, phospho-mTOR (Ser2448; Catalog No. 5536, Cell Signaling) at 1:500, total-S6 ribosomal protein (Catalog No. 2317, Cell Signaling) at 1:1000, phospho-S6 ribosomal protein (Ser235/236; Catalog No. 4858, Cell Signaling) at 1:500 and GAPDH (Catalog No. ab8245, Abcam, Cambridge, UK) at 1:20,000.

Plasma and cardiac BCAA levels were measured from plasma and heart samples, respectively, using a colorimetric assay kit (Catalog No. ab83374, Abcam, Cambridge, UK) conforming to the manufacturer instructions.

**Cardiac RNA isolation, RNA sequencing, and sequencing analysis**

RNA isolation, RNA sequencing, and sequencing analysis were performed as described previously.[8] Briefly, frozen LV tissue was homogenized, and mRNA was extracted with the RNeasy Universal Mini Kit (Qiagen). The Boston University Microarray and Sequencing Resource Core Facility performed RNA quantity and quality measurements, library preparations, RNA sequencing, and analyses. Sequencing libraries were generated using the NEBNext Ultra II RNA kit with poly(A) selection and run on an Illumina NextSeq 2000 platform with 100x100bp paired-end reads. FASTQ files were aligned to mouse genome build mm10 using STAR (version 2.6.0c), and assessed for quality using FastQC (version 0.11.7) and RSeQC (version 3.0.0). Ensembl-Gene–level counts for nonmitochondrial genes were generated using featureCounts (Subread package, version 1.6.2) and Ensembl annotation build 100 (uniquely aligned proper pairs, same strand). Human homologs of mouse genes were identified using HomoloGene (version 68). Gene Set Enrichment Analysis (GSEA) (version 2.2.1) was used to identify biological terms, pathways, and processes that are coordinately upregulated or downregulated within each pairwise comparison. Gene versions of the Hallmark, Biocarta, KEGG, PID, Reactome, WikiPathways, Gene Ontology (GO), and

transcription factor and microRNA motif gene sets obtained from the Molecular Signatures Database (MSigDB), version 7.5.1.

### Cardiac proteomics and phosphoproteomics

Cardiac proteomics and phosphoproteomics were performed as described previously.[24] Frozen LV samples were placed in lysis buffer containing protease (Sigma) and phosphatase inhibitor (PhosSTOP Roche, MilliporeSigma). Afterwards, they were homogenized and sonicated and digested with sequencing-grade Trypsin (1:50 enzyme to protein ratio, w/w, ThermoFisher). Peptide digests were de-salted using a C18 Sep-Pak (Waters) according to the manufacturer's instructions, resuspended in 100 mM TEAB and quantified by Quantitative Colorimetric Peptide Assay (Pierce) prior to tandem mass tag (TMT) labelling. 100 µg of peptide digest was mixed with a unique amine-reactive isotope-coded isobaric tandem mass tag (TMT-16-plex) reagent (ThermoFisher). After pooling, labeled peptide was desalted, dried, and suspended in buffer (0.1% ammonium hydroxide, 2% acetonitrile). The pooled sample mixture was pre-fractionated by high pH reversed-phase HPLC on a XBridge Peptide BEH C18 column (Waters) using an Agilent 1100 HPLC system. Peptides were eluted using a gradient of mobile phase A (0.1% NH4OH, 2% ACN) to B (0.1% NH4OH, 98% ACN) over 48 min, and collected as 12 pooled fractions. For phosphoproteomics, the bulk (95%) of each sample was subjected to phosphopeptide enrichment using $FeO_2$ metal-chelate resin (PureCube Fe-NTA MagBeads, Cube Bio-tech), while the remaining (5%) portions were analyzed directly by nanoflow LC/MS as bulk proteome measurements. Stable isotope-labeled peptides were reconstituted in mobile phase A (0.1% formic acid, 2% acetonitrile) prior to LC/MS analysis. After loading onto a C18 reverse-phase pre-column (ThermoScientific), peptides were gradient separated on an EASY-Spray C18 nanocolumn (ThermoScientific) and electro-sprayed at ~250 nL/min into the Exploris instrument operated in positive ion mode. Data-dependent MS/MS spectra were acquired automatically to select the 12-most abundant peptides for MS/MS analysis with fragmentation

by high energy dissociation. The resulting RAW files were searched by MaxQuant (1.6.7.0) using default settings against the mouse proteome. Bioinformatic analysis was performed using open-source R software. A total of 6,548 unique proteins and 5,068 unique phosphosites were identified in this study.

### Contractility and $Ca^{2+}$ handling in isolated cardiomyocytes

Left ventricular cardiomyocytes were isolated, and cell contractility and $Ca^{2+}$ handling was measured as we described previously.[25–27] Briefly, cardiomyocytes were isolated from the left ventricle enzymatically, placed in physiological Tyrode solution [containing 137 mM NaCl, 5.4 mM KCl, 1.2 mM $CaCl_2$, 0.5 mM $MgCl_2$, 10 mM HEPES, 5 mM glucose, and 0.5 mM probenecid; pH 7.40] and externally paced at 5 Hz at 37°C. Cardiomyocyte contractility and intracellular $Ca^{2+}$ levels (using fura-2 AM, Molecular Probes, Invitrogen, Waltham, MA, USA) were measured simultaneously using an integrated system (IonOptix, featuring a HyperSwitch dual 340- to 380-nm excitation light source). Probenecid was added to the superfusing solution to increase fura-2 retention. Sarcomere shortening was expressed as the percentage of diastolic sarcomere length. The time constant ($Tau_{Ca}$) of a monoexponential curve fitted with fura ratio decrease in diastole was measured to determine SERCA activity. Diastolic sarcomere length and diastolic $Ca^{2+}$ levels were measured just before the following $Ca^{2+}$ transient.

### Cardiac glucose metabolism

To determine cardiac glucose metabolism, the metabolic fate of glucose was tracked as described previously.[28] Mice were administered 300 mg of $^{13}C$-labelled glucose (CLM-1396-25, Cambridge Isotope Laboratories, Tewksbury, MA, USA) by oral gavage after 12-h fasting. Heart was harvested 90 min after oral gavage. Cardiac labelled glucose metabolism was analyzed by liquid chromatography–mass spectrometry (LC-MS) as described previously.[28]

**Blood glucose, lactate and ketone levels**

Blood glucose, lactate and ketone (beta-hydroxybutyrate) levels were measured by handheld meters using specific test strips conforming to the manufacturer's instructions and as described previously in studies with mice. Blood glucose was measured by an AimStrip Plus glucometer (Germaine Laboratories, San Antonio, TX, USA),[8] blood lactate was measured by Lactate Plus blood lactate meter (Nova Biomedical, Waltham, MA, USA),[29] and blood ketone (beta-hydroxybutyrate) level was measured by Precision Xtra glucose and ketone monitoring system (Abbott Laboratories, Abbott Park, IL, USA).[30]

**Human induced pluripotent stem cell (hiPSC)-derived cardiomyocyte cell culture**

Human iPSC-derived cardiomyocytes were generated from the Harvard Personal Genome Project line 1 (PGP1, GM23338), generously provided by the Seidman Lab at Harvard Medical School.[31,32] Heterozygous mutations were introduced into the MYH7 allele in hiPSC as described previously.[31] hiPSCs were cultured with mTESR Plus media (STEMCELL Technologies, Vancouver, BC, Canada) on Matrigel (Thermo Fisher Scientific, Waltham, MA, USA)-coated plates and differentiated into cardiomyocytes (day 0) at 80–100% confluency via activation of the WNT pathway with 8 μM CHIR 99021 (Tocris, Bristol, UK) in RPMI + GlutaMAX media supplemented with B27 minus insulin (RPMI and B27 minus, Thermo Fisher Scientific). After 24 h, the cells were washed with PBS and given RPMI and B27 minus. On day 3, the cells were switched to CDM3 media consisting of RPMI supplemented with 500 μg/ml bovine serum albumin and 213 μg/ml L-ascorbic acid 2-phosphate trisodium salt (Sigma Aldrich). The CDM3 media was supplemented with 5μM IWP-4 (Tocris) and 1μM retinol inhibitor (BMS 453, Cayman Chemical). On day 5, the cells were given CDM3 supplemented with 1 μM retinol inhibitor. On day 7, the media was replaced with fresh CDM3. On day 9 and 11, the cardiomyocyte population was purified by metabolic selection via RPMI glucose-free media (Gibco, Waltham, MA, USA) supplemented with 4 mM of DL-lactate (MilliporeSigma,

Burlington, MA, USA). On day 13, the media was replaced with CDM3. Following 24h, cells were replated onto fibronectin-coated tissue culture plastic plates using 0.25% Trypsin-EDTA (Thermo Fisher Scientific) and 10 µg/mL deoxyribonuclease I (STEMCELL Technologies). They were then placed in RPMI B27+ supplemented with 5 µM Y-27632 (Tocris) and 2% fetal bovine serum (MilliporeSigma) for seeding. Cultures were maintained on RPMI + GlutaMAX media supplemented with B27 plus insulin. Isogenic healthy wild-type (WT) hiPSC-CMs or MYH7-variant (R403Q+/-) hiPSC-CMs were differentiated in time-matched batches. On day 16+ of differentiation, WT and mutant hiPSC-CMs were plated at 180k cells/well in a 6-well plate. Two days after plating, cells were treated with vehicle (dimethyl sulfoxide) or 1 µM empagliflozin for 24 hours. Empagliflozin concentrations were selected following plasma concentrations of the drug in patients described previously.[33,34] Three days after plating, the hiPSC-CMs were washed three times in Tyrode's buffer (Thermo Fisher Scientific) and incubated in 10 µM Rhod-3 AM calcium indicator, PowerLoad, and Probenecid for 45 min covered from light, following the protocol from the Rhod-3 AM calcium imaging kit (ThermoFisher). Cells were washed in Tyrode's buffer and incubated in Probenecid for an additional 45 min. Cells were then washed in Tyrode's buffer three additional times and kept in Tyrode's during live imaging. Calcium transients were acquired at 30 frames per second at 15X on a Nikon Eclipse Ti (Nikon Instruments, Tokyo, JP) with an Evolve EMCCD Camera (Photometrics, Tucson, AZ, USA), equipped with a temperature and CO2 equilibrated environmental chamber. hiPSC-CMs were electrically stimulated at 1 Hz using a C-Pace EP stimulator (IonOptix, Westwood, MA, USA). Videos were acquired at 15 locations per experimental group with a 560 nm laser illumination wavelength. Calcium transients of each cell were calculated using a custom Matlab script tracking intensity change over time within the cell. This was repeated over three differentiations (N = 3). The relaxation rate was calculated as change in fluorescence and the T75 relaxation during each beat. All data were normalized to the average WT values for the matched differentiation.

**Single-nucleus RNA sequencing analysis of cardiac samples**

Nuclei were isolated from 50 mg fresh frozen cardiac samples (n=2) using the 10x Genomics Chromium Nuclei isolation kit (PN-1000494, with RNAse inhibitor; 10X Genomics, Pleasanton, CA, USA), conforming to the manufacturer protocol. Nuclei integrity and counts were determined using a hemocytometer. Single nuclei sequencing libraries were generated according to 10X Genomics' Chromium Single Cell 3' GEM, Library & Gel Bead Kit by the Boston University Single Cell Sequencing Core conforming to the manufacturer's protocol and as described previously.[35–38] Briefly, to generate Gel Bead in Emulsion (GEMs), nuclei suspension was combined with 3' Gel Beads containing unique bead barcodes and transcript UMIs and loaded to Chromium Chip G. 3,000 nuclei per sample with a range of 77–83% integrity was targeted for capture and library preparation (around 10,000 nuclei per sample was captured). Barcoded, full-length cDNA, was generated by incubation and dissolving the Gel Bead from GEMs. Pooled fractions were amplified via PCR to generate mass sufficient for library preparation. Double-stranded cDNA was processed according to the manufacturer's protocol (10X Genomics, Pleasanton, CA, USA). cDNA libraries were assessed for size distribution and molarity via the Bioanalyzer High Sensitivity DNA Assay (Agilent Technologies, Lexington, MA, USA). Libraries were sequenced using an Illumina NextSeq 2000 instrument (Illumina, San Diego, CA, USA). The run generated over 1.2 billion reads with 94% > Q30 and an error rate of 0.15%. Bioinformatics analysis was performed by the Boston University Single Cell Sequencing Core as described previously.[35–38] Briefly, Uniform Manifold Approximation and Projection (UMAP)/Cellular Latent Dirichlet Allocation (celda) analysis identified 9 cell types and 15 cell clusters. Gene set enrichment analysis (GSEA) using fgsea was performed to identify coordinately regulated pathways in fibroblasts. The fgsea package was used to identify enrichment of pathways in the Molecular Signatures Database (MSigDB), version 7.5.1. The gene set collections used were the Hallmark, Reactome, KEGG, WikiPathways, and Gene Ontology sets. Pathway enrichment was tested by generating differentially expressed genes between samples, with FDR < 0.05 as a threshold. The pathways were

sorted by enrichment score, and the top and bottom 25 were selected by the p-values. More details on the single-nucleus RNA sequencing analysis can be found in datasets deposited to Gene Expression Omnibus (GEO).

**Statistical analysis**

Results are presented as mean±SEM (unless stated otherwise in figure legend). One-way ANOVA with Bonferroni multiple comparisons test (adjusted p-value threshold <0.05) was used to analyze the $^{13}C$ and $^{23}Na$ NMR data (glucose uptake, substrate oxidation rates, lactate production and intracellular sodium; Figure 3 and S6), energy cost of contraction at LW and HW (Figure 4H), echocardiography and histology data (Figure 5, S1, S8 and S10), and western blot, cardiac BCAA levels and heart weight data (Figure 6 and Table S4), echocardiography and histology data in the experiment with mice harboring cardiac troponin R92L mutation (Figure S12), echocardiography data in the experiment with BT2 administration (Figure S17), cardiac levels of individual BCAAs measured by liquid chromatography-mass spectrometry (Figure S14), plasma BCAA and blood glucose, ketone and lactate levels (Figure S16), cardiac levels of carnitines as measured by liquid chromatography-mass spectrometry (Figure S7), and sarcomere length, shortening and relaxation and calcium transient in isolated cardiomyocytes (Figure S15). Two-way repeated measures ANOVA with Bonferroni multiple comparisons test (adjusted p-value threshold <0.05) was used to analyze the contractile function and $^{31}P$ NMR data from the isolated beating hearts (Figure 4A through 4G). Two-way repeated measures ANOVA with Tukey's multiple comparisons test was used to analyze longitudinal echocardiography data on EMPA's effect on advanced HCM phenotype (Figure S11). Two-way ANOVA with Tukey's multiple comparisons test was used to analyze carnitine isotopologues (Figure S7). Two-way ANOVA with a mixed-effect model that corrected for multiple comparisons with a Bonferroni test (adjusted p-value threshold <0.05) was used to analyze calcium transient and relaxation rate data in iPSC-CMs (Figure S18). Specific statistical tests and n per group are indicated in figure legends. The statistical analyses were

performed using GraphPad Prism 10 (GraphPad Software, La Jolla, CA). P<0.05 was considered significant.

For proteomics and phosphoproteomics data analysis (Figure 2 and S5, Table S3), peptide feature signal intensities were log transformed and loess normalized. LIMMA R package was used for differential analysis (moderated Student t-tests), and to generate ranked lists for subsequent enrichment analysis using the Benjamini-Hochberg false discovery rate (FDR) correction.[39] Statistical enrichment analysis was performed using fgsea R package with the Molecular Signatures Database (MSigDB) version 7.5.1.[40] Pathways with FDR <0.05 in the R403Q-CD comparison was considered significant. Volcano plots were created with the EnhancedVolcano R package.[41]

For RNA sequencing data analysis (Table 1, S1 and S2), variance-stabilizing transformation (VST) was accomplished using the Variance Stabilizing Transformation function in the DESeq2 R package (version 1.32.0). Differential expression was assessed using the Wald test implemented in the DESeq2 R package. Correction for multiple hypothesis testing was accomplished using the Benjamini-Hochberg false discovery rate (FDR). FDR <0.05 was considered significant. All analyses were performed using the R environment for statistical computing (version 4.1.2). Similar gene set enrichment analysis (GSEA) using fgsea with the Molecular Signatures Database (MSigDB) version 7.5.1 was performed for single-nucleus RNA sequencing data, with FDR < 0.05 as a threshold (Figure S2 and S3).

**Dataset deposition**

The RNA sequencing datasets have been deposited in Gene Expression Omnibus (GEO Series ID: GSE270093 for tissue RNAseq and GEO Series ID: GSE283722 for single-nucleus RNAseq). The mass spectrometry proteomics and phosphoproteomics data have been deposited to the ProteomeXchange Consortium via the PRIDE (Proteomics Identifications Database) partner repository with the dataset identifier PXD053046 and

10.6019/PXD053046. The data that support the findings of this study are available from the corresponding author upon reasonable request.

**Supplementary Figures**

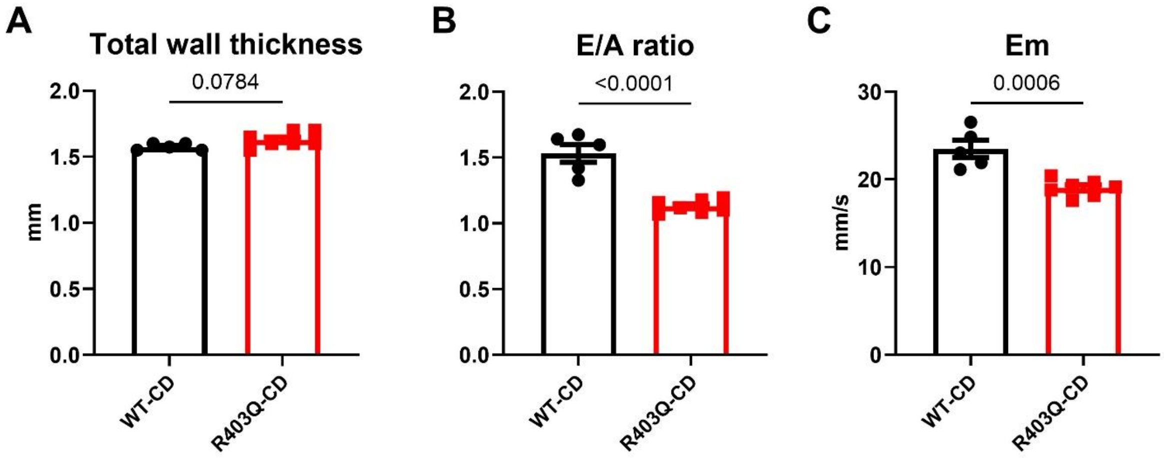


**Figure S1. 12-week-old R403Q mice show LV diastolic dysfunction without LV hypertrophy.** Echocardiography of 12-week-old R403Q mice shows LV diastolic dysfunction as evidenced by decreased E/A ratio (**B**) and Em (**C**) without LV hypertrophy (**A**). Data shown are mean±SEM. n=5-7. *P* values were obtained by unpaired t test.

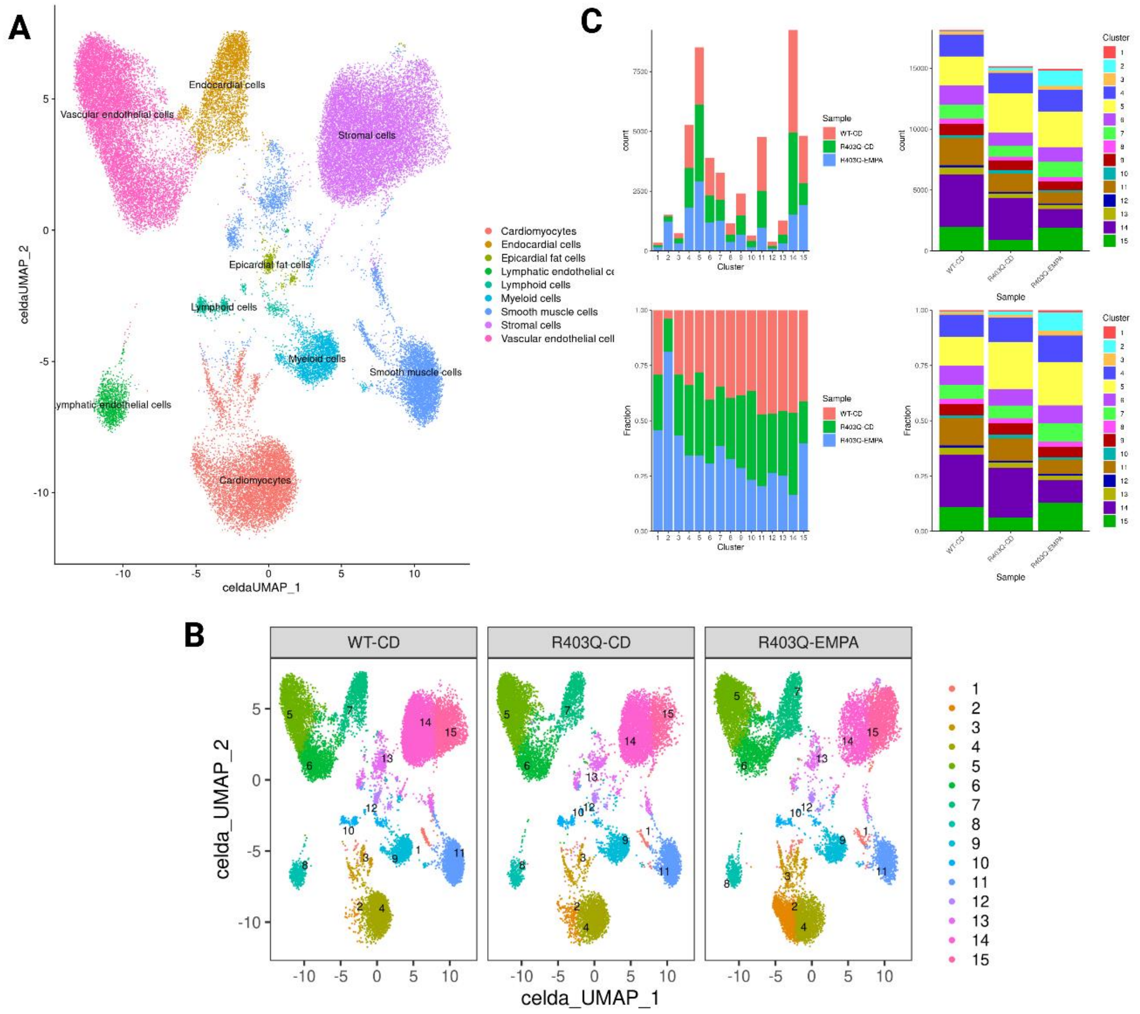

**Figure S2. Cell clusters in cardiac tissue as identified by single-nucleus RNA sequencing analysis.** Uniform Manifold Approximation and Projection (UMAP)/Cellular Latent Dirichlet Allocation (celda) analysis identified 9 cell types and 15 cell clusters. Pooled nuclei cell type distribution (**A**), the distribution of 15 identified clusters in each study groups (**B**) and absolute and proportional cluster abundance (**C**) are shown. Fibroblasts are the predominant cell type amongst Stromal cells identified. 2 cardiac samples per each study group were analyzed.

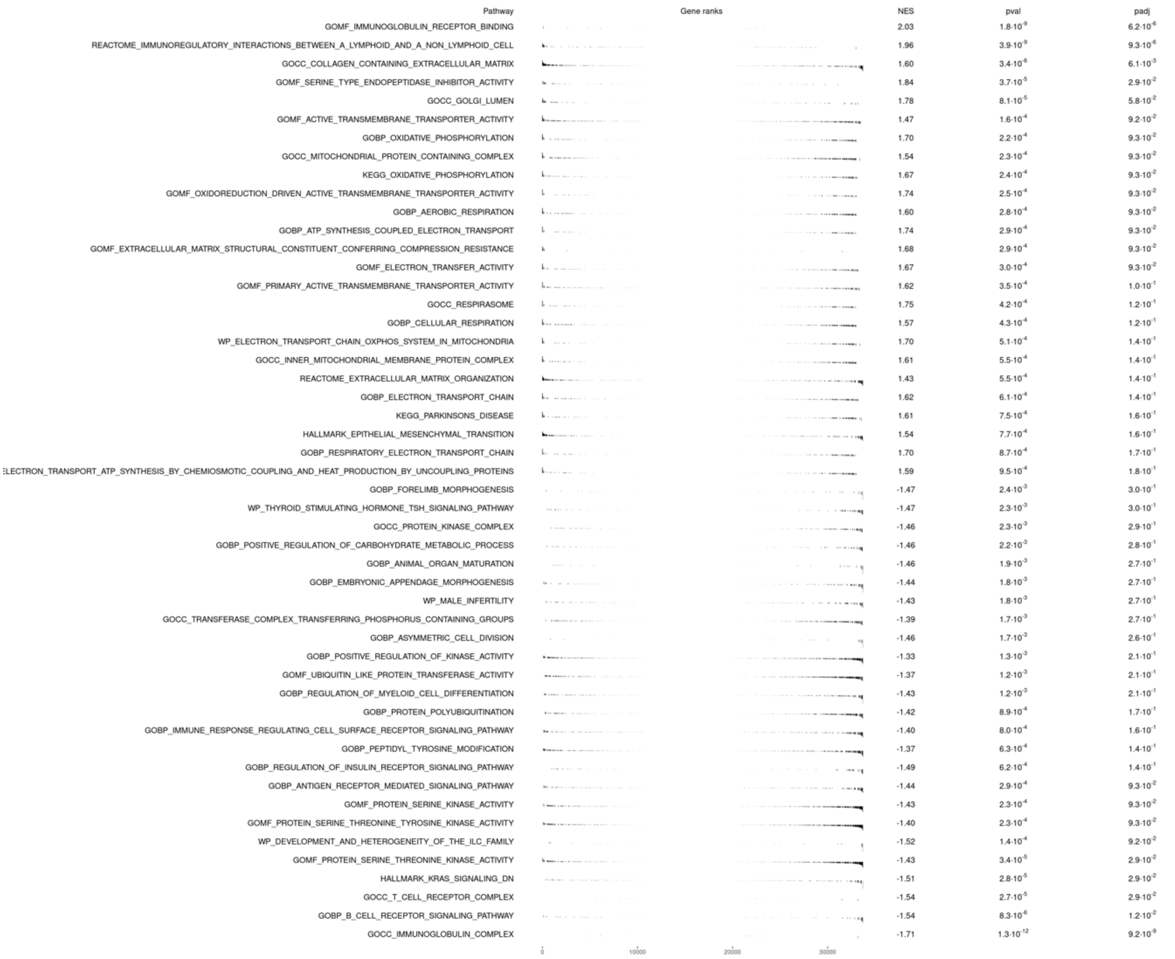


**Figure S3. Top 25 most up- or down-regulated pathways in fibroblasts from R403Q-CD vs. WT-CD hearts.** n = 2. NES, Normalized Enrichment Score; p, nominal p value; padj, adjusted p value; GOBP, Gene Ontology Biological Process; GOCC, Gene Ontology Cellular Component; GOMF, Gene Ontology Molecular Function; KEGG, Kyoto Encyclopedia of Genes and Genomes; WP, WikiPathways. Pathway enrichment was tested by generating differentially expressed genes between samples, with FDR < 0.05 as a threshold. Adjusted p-value < 0.05 is considered significant.

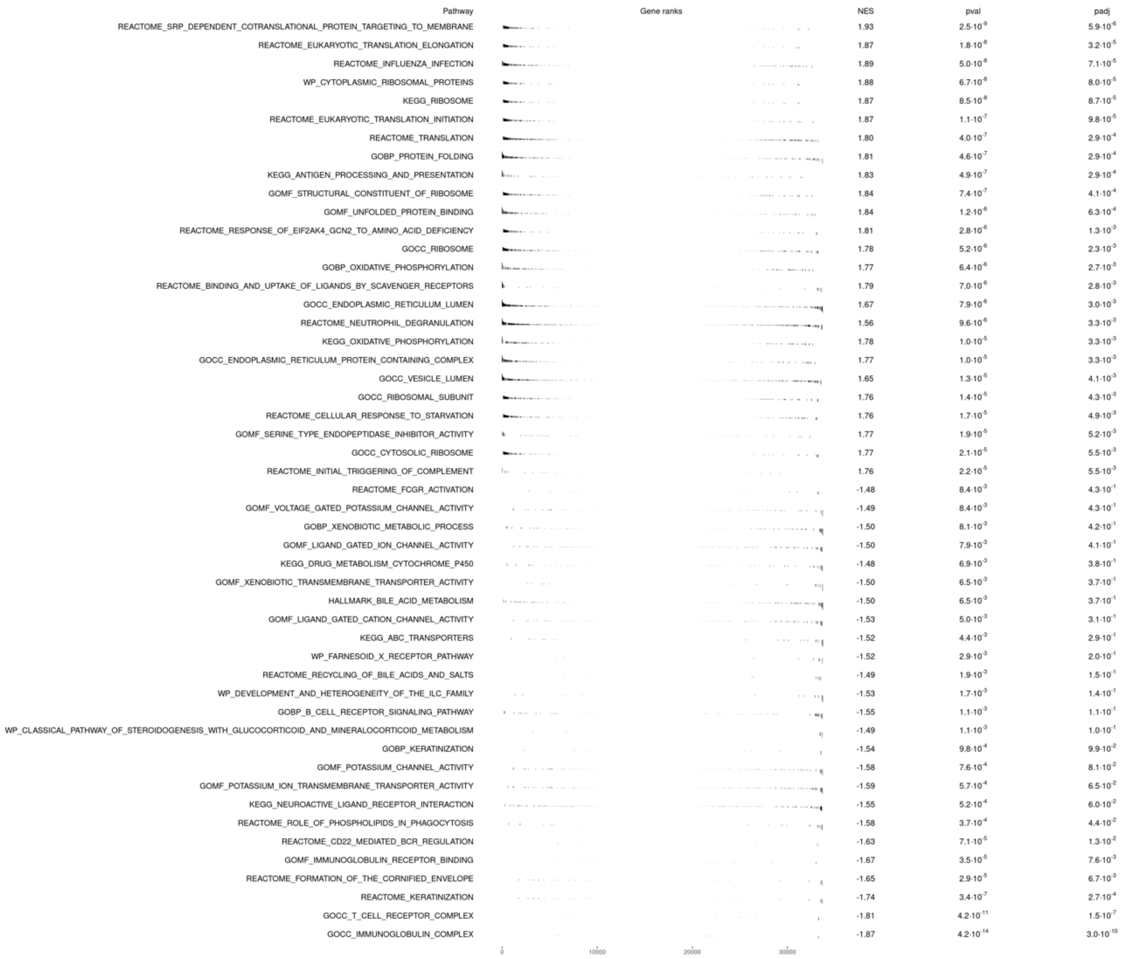


**Figure S4. Top 25 most up- or down-regulated pathways in fibroblasts from R403Q-EMPA vs. R403Q-CD hearts.** n = 2. NES, Normalized Enrichment Score; p, nominal p value; padj, adjusted p value; GOBP, Gene Ontology Biological Process; GOCC, Gene Ontology Cellular Component; GOMF, Gene Ontology Molecular Function; KEGG, Kyoto Encyclopedia of Genes and Genomes; WP, WikiPathways. Pathway enrichment was tested by generating differentially expressed genes between samples, with FDR < 0.05 as a threshold. Adjusted p-value < 0.05 is considered significant.

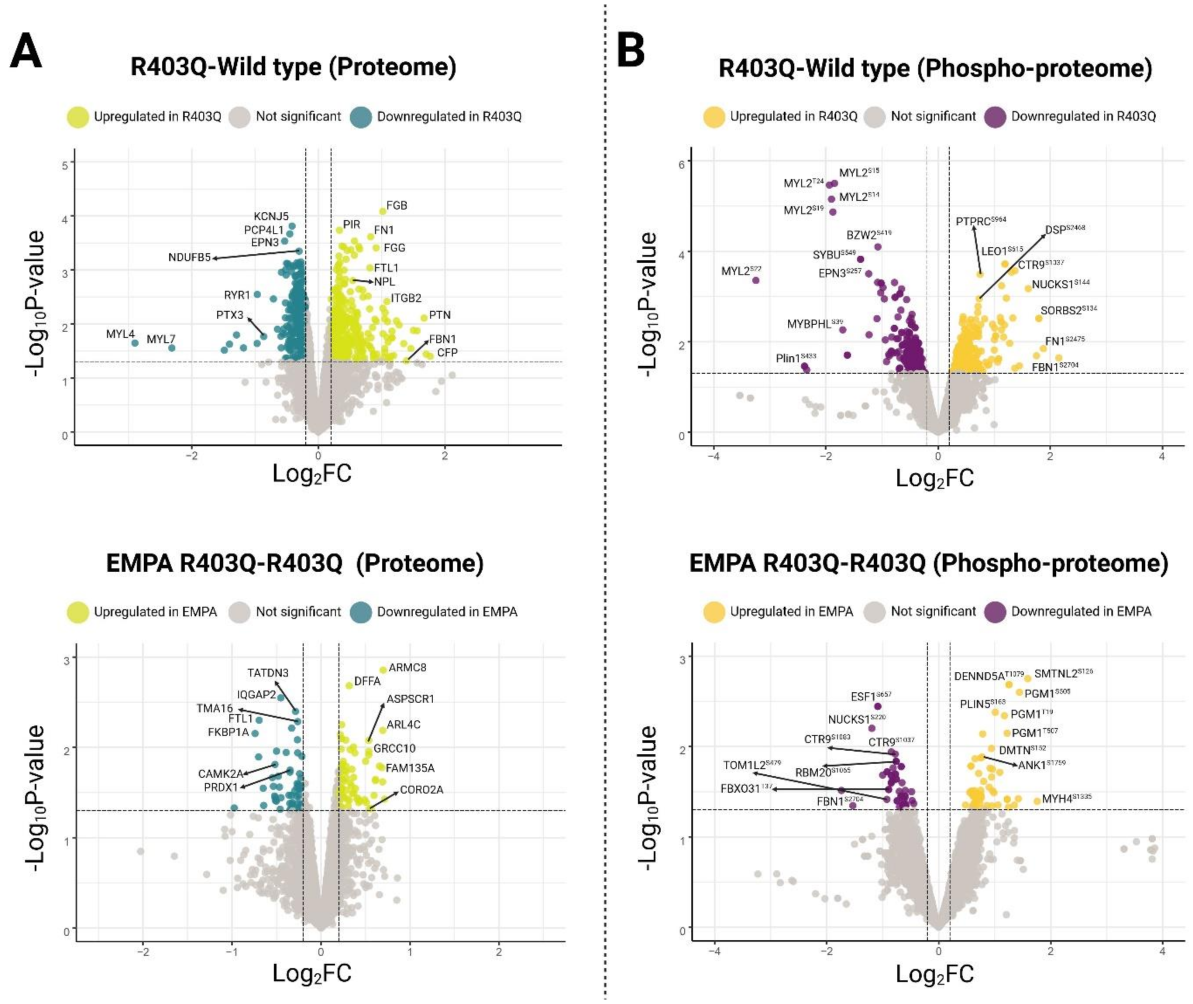


**Figure S5. Cardiac proteomic and phosphoproteomic analysis performed in 25-week-old mice after 16 weeks of treatment.** Volcano plots of differentially expressed proteins (**A**) and phosphor-proteins (**B**) show negative log-transformed p-values against $\log_2$-transformed fold-change ($\log_2$FC) measured between specimens. Non-grey values are highlighted based on a threshold of p-value<0.05 and $\log_2$FC ≥0.2 or ≤-0.2.

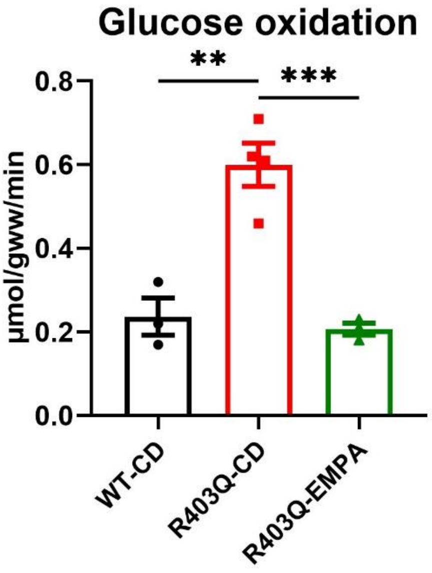


**Figure S6. Empagliflozin normalizes glucose oxidation rate in R403Q hearts.** The glucose oxidation rate was elevated 2.6-fold in R403Q hearts vs WT and EMPA normalized it. Data shown are mean±SEM. n=3-4. *P* values were obtained by one-way ANOVA with Bonferroni multiple comparisons tests (adjusted p-value threshold <0.05). ***P*<0.01 and ****P*<0.001.

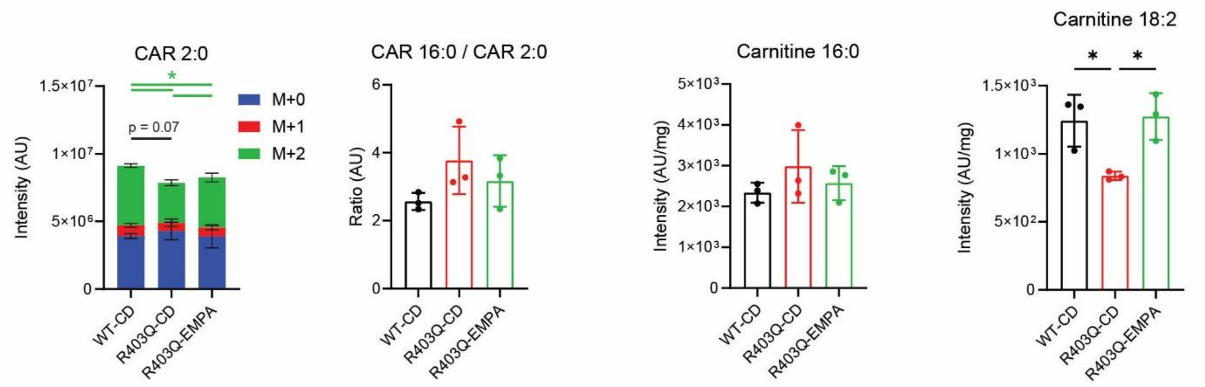


**Figure S7. Cardiac levels of carnitines as measured by liquid chromatography-mass spectrometry.** Acetylcarnitine serves as a proxy to evaluate the balance between acetyl-CoA flux from glycolysis and mitochondrial β-oxidation. In wild-type hearts, $^{13}$C-labeled glucose contributed to acetylcarnitine, reflected as the M+2 isotopologue (green bar). In R403Q hearts, while total acetylcarnitine levels remained unchanged, the proportion of the M+2 isotopologue was reduced, indicating that fewer $^{13}$C carbon atoms from tracer glucose were directed toward oxidative phosphorylation. Treatment with empagliflozin partially restored this rewiring. The ratio of palmitoylcarnitine (CAR 16:0) to acetylcarnitine (CAR 2:0) serves as a marker of effective β-oxidation. An increasing trend in this ratio was observed in R403Q hearts, suggesting impaired β-oxidation and accumulation of acylcarnitine intermediates. While palmitoylcarnitine levels showed an increasing trend, linoleoylcarnitine (CAR 18:2), a major essential fatty acid substrate, was decreased in R403Q hearts, indicating an inability to utilize circulating linoleic acid. Empagliflozin restored linoleoylcarnitine levels, suggesting improved utilization of essential fatty acids. This differential behavior between palmitoylcarnitine (an endogenous metabolite) and linoleoylcarnitine (an essential fatty acid) may reflect a shift toward enhanced utilization of exogenous fatty acids from circulation rather than endogenous stores. Taken together, these findings suggest that empagliflozin normalizes dysregulated acyl-carnitine profiles and improves fatty acid oxidation (FAO) in R403Q hearts. AU, arbitrary unit. Data are means ± SD, n = 3. One-way ANOVA (groups, Bonferroni's multiple comparisons test with adjusted p-value threshold <0.05), * statistically significant at $p < 0.05$. Isotopologues: Two-way ANOVA (group, isotopologue, Tukey's multiple comparisons test)

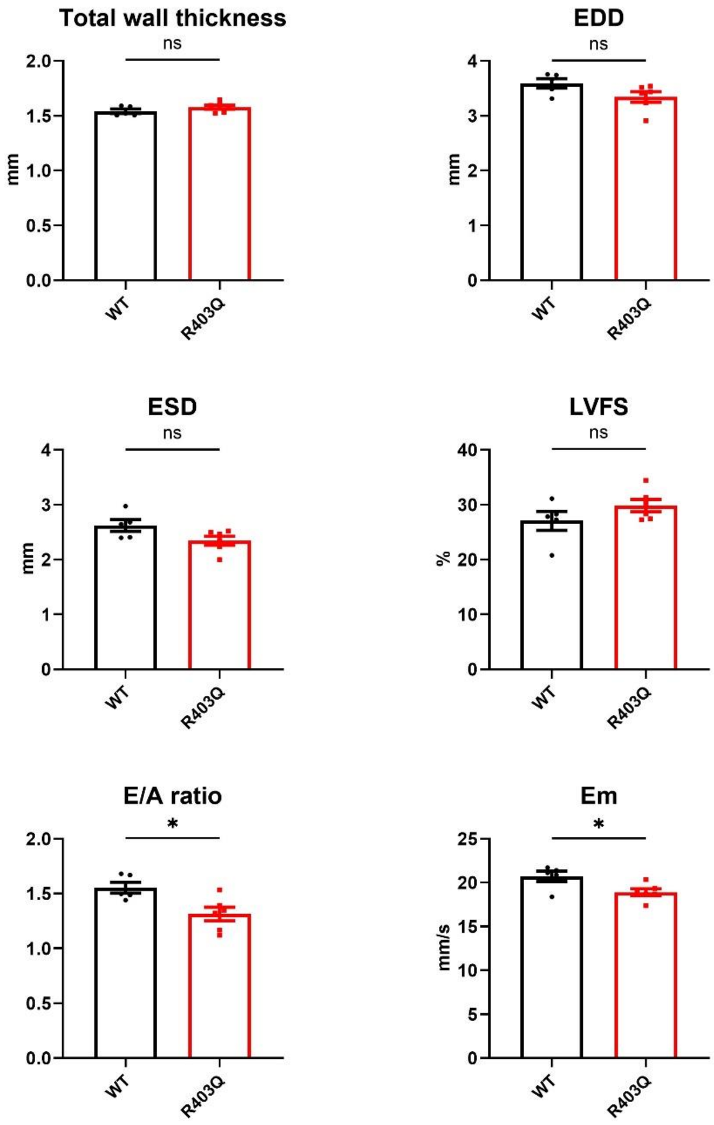


**Figure S8. Echocardiography of 9-10-week-old R403Q mice.** Mice harboring myosin R403Q mutation present with diastolic dysfunction but no cardiac hypertrophy at the age of treatment initiation. Left ventricular end-diastolic (EDD) and end-systolic diameter (ESD), fractional shortening (LVFS). E/A ratio, ratio of peak early (E) and late (A) mitral inflow velocities; Em, tissue Doppler of myocardial peak early diastolic velocity. Data shown are mean±SEM. n=5-6. *P* values were obtained by unpaired t test. **P*<0.05, ns, not significant.

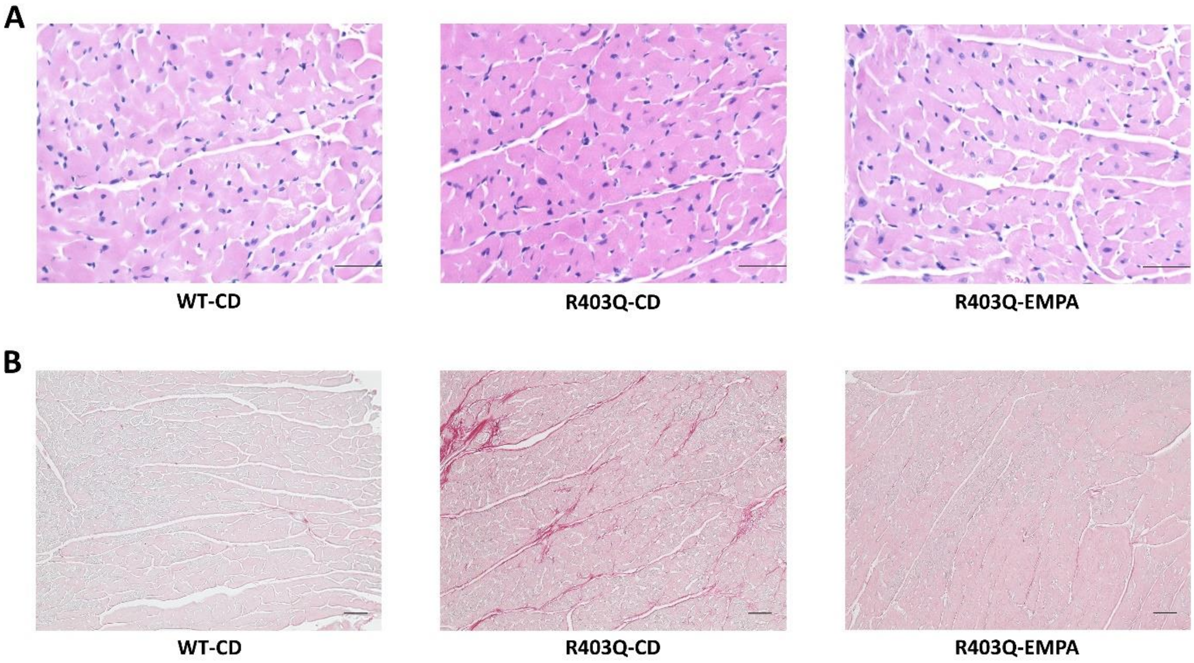


**Figure S9. Additional histopathological images depicting effect of EMPA on cardiac hypertrophy and fibrosis in R403Q hearts.** A 16-week EMPA treatment reduced cardiac hypertrophy (**A**) and fibrosis (**B**) in R403Q hearts. Representative photomicrographs, hematoxylin-eosin for myocyte cross-section area analysis (**A**) or picrosirius red for cardiac fibrosis analysis (**B**), transmitted light, scale bar indicates 50 µm.

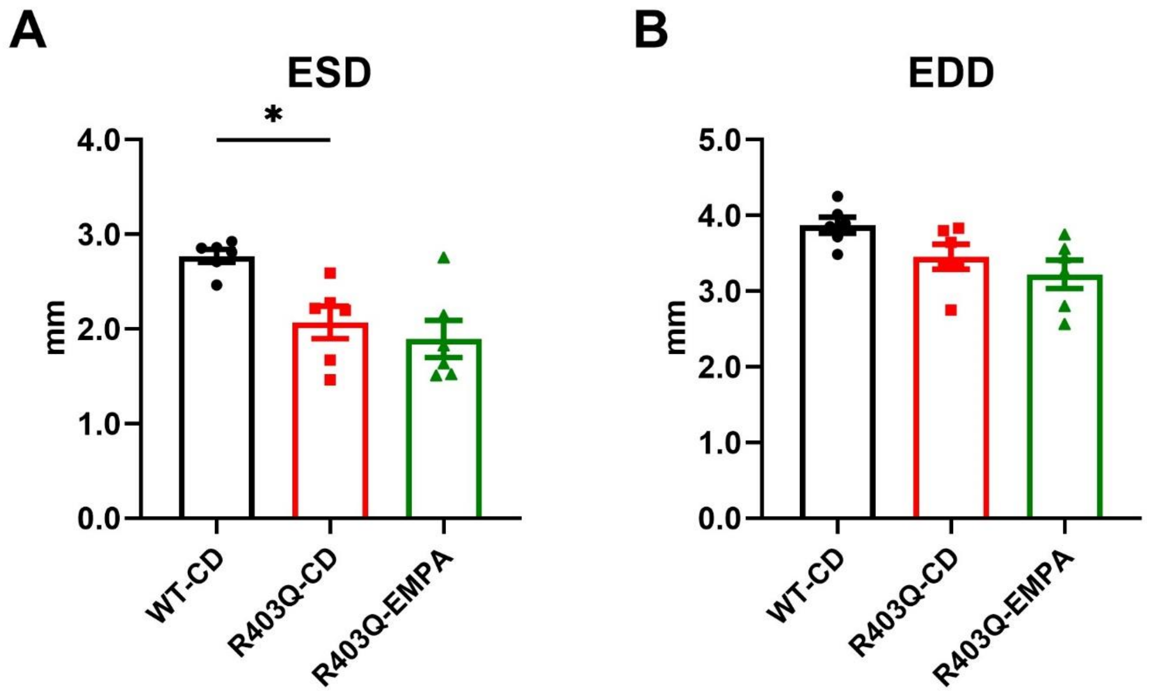


**Figure S10. Heart dimensions after 16-week EMPA treatment as assessed by echocardiography.** R403Q hearts showed decreased end-systolic diameter (ESD) (**A**) and non-significatly decreased end-diastolic diameter (EDD) (**B**). 16-week EMPA treatment did not affect these measurements. Data shown are mean±SEM. n=6. *P* values were obtained by one-way ANOVA with Bonferroni multiple comparisons tests (adjusted p-value threshold <0.05). **P*<0.05.

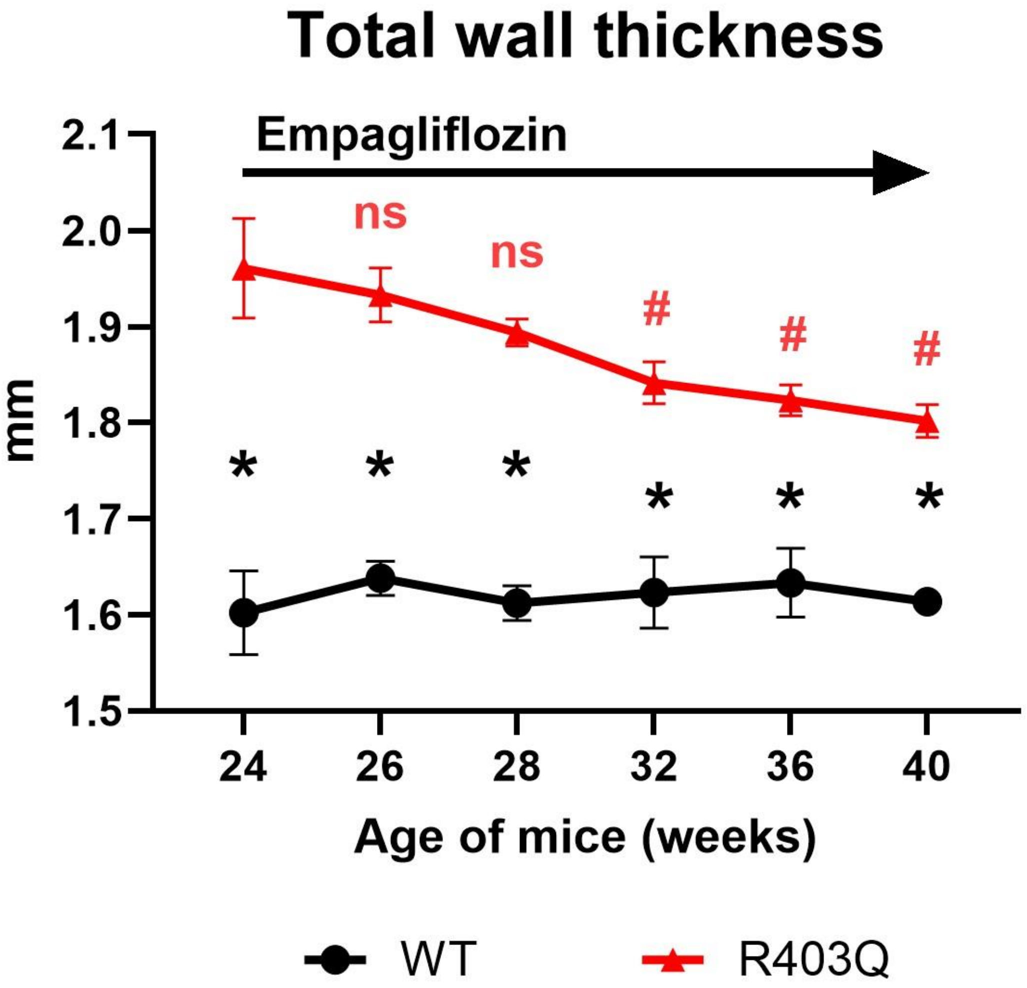


**Figure S11. Empagliflozin reverses cardiac hypertrophy in R403Q hearts if treatment initiated at advanced HCM phenotype**. A 16-week EMPA treatment was initiated in R403Q mice aged 24 weeks presenting with developed HCM phenotype. Total wall thickness was assessed longitudinally by echocardiography. Left ventricular hypertrophy was significantly reduced after 8 weeks of EMPA treatment and steadily declined further till the end of the study at 16 weeks of treatment. Data shown are mean±SEM. n=4-5. *P* values were obtained by two-way repeated measures ANOVA with Tukey's multiple comparisons tests. **P*<0.05 vs WT, # *P*<0.05 vs R403Q at 24-week-old; ns, not significant.

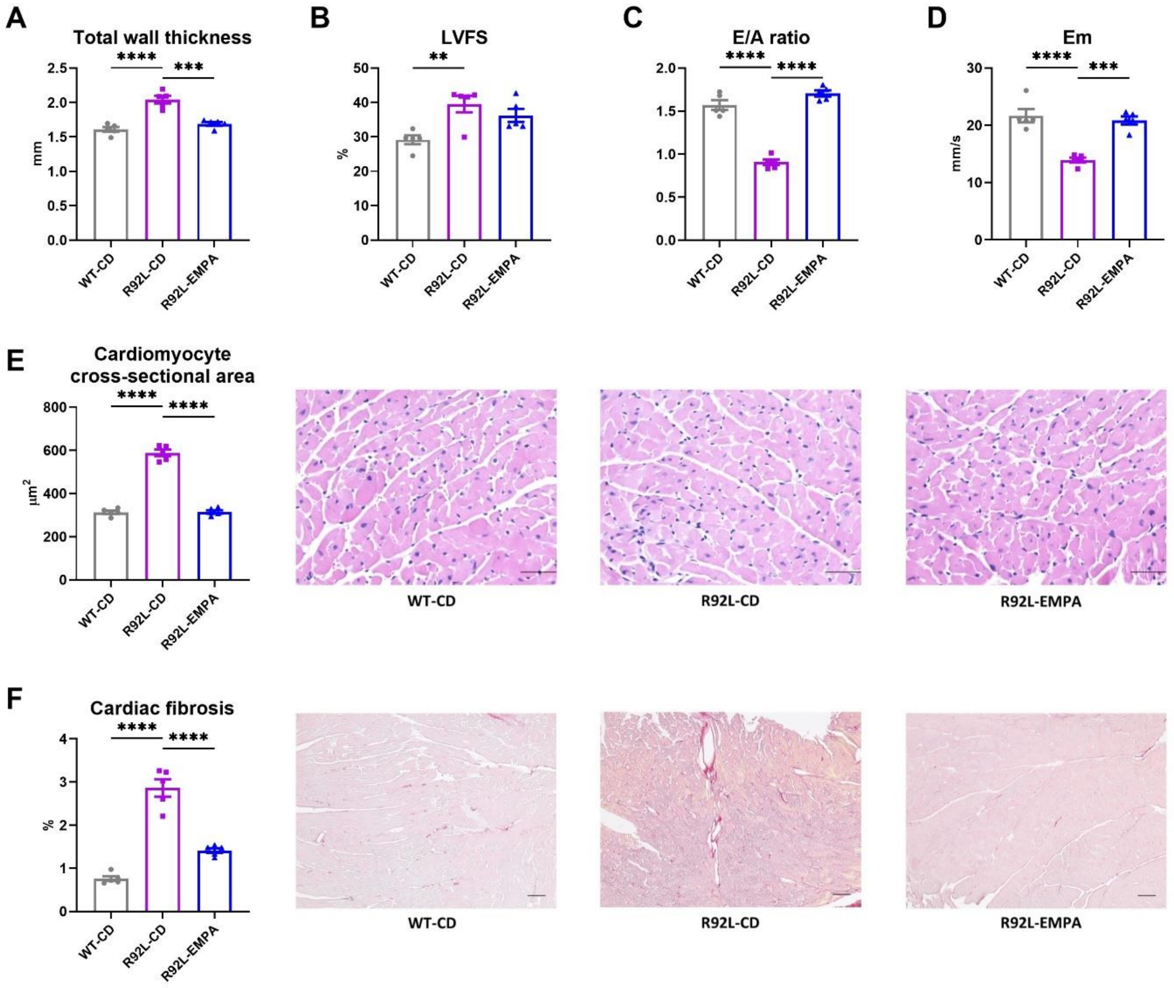


**Figure S12. Empagliflozin prevents cardiac hypertrophy and improves diastolic function in R92L hearts.** R92L hearts developed LV hypertrophy as evidenced by increased total wall thickness (**A**) on echocardiogram and increased cardiomyocyte cross-section area (**E**) and fibrosis (**F**) in histopathology. Hypercontractility in R92L hearts was evidenced by increased LV fractional shortening (LVFS) (**B**). Furthermore, deteriorated LV diastolic function was evidenced by reduced E/A ratio (**C**) and Em (**D**). A 16-week EMPA treatment reduced cardiac hypertrophy and fibrosis (**A, E, F**). Additionally, EMPA enhanced LV diastolic function (**C** and **D**) without affecting hypercontractility (**B**). Data shown are mean±SEM. n=5. *P* values were obtained by one-way ANOVA with Bonferroni multiple comparisons tests (adjusted p-value threshold <0.05). $^{**}P<0.01$, $^{***}P<0.001$, and $^{****}P<0.0001$. Representative photomicrographs, hematoxylin-eosin for myocyte cross-section area analysis (**E**) or picrosirius red for cardiac fibrosis analysis (**F**), transmitted light, scale bar indicates 50 μm.

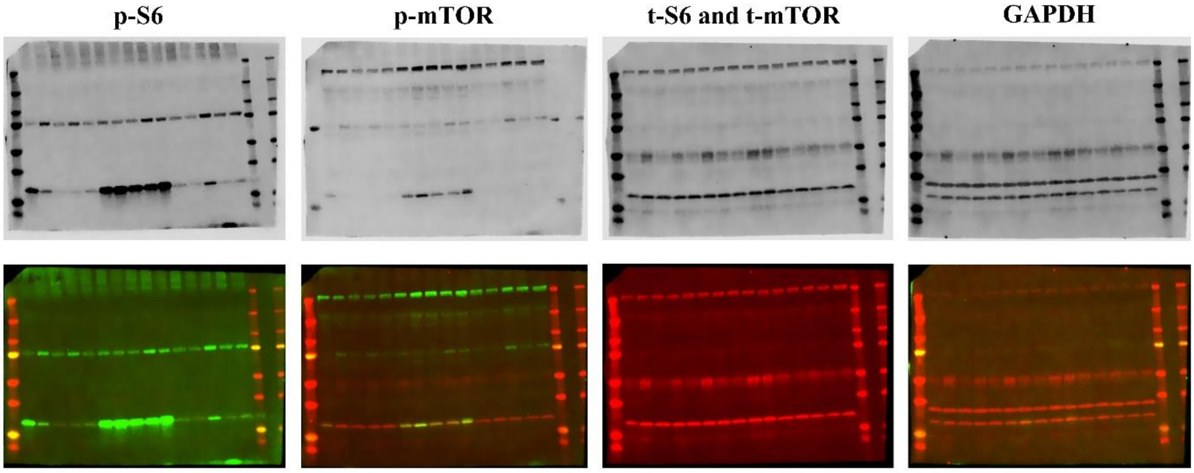


**Figure S13. Representative uncropped western blot images.** Phospho-S6 and total-S6 ribosomal protein, phospho-mTOR and total-mTOR, and GAPDH proteins. Left-to-right, WT-CD: blots 1-5, R403Q-CD: blots 6-10, R403Q-EMPA: blots 11-15.

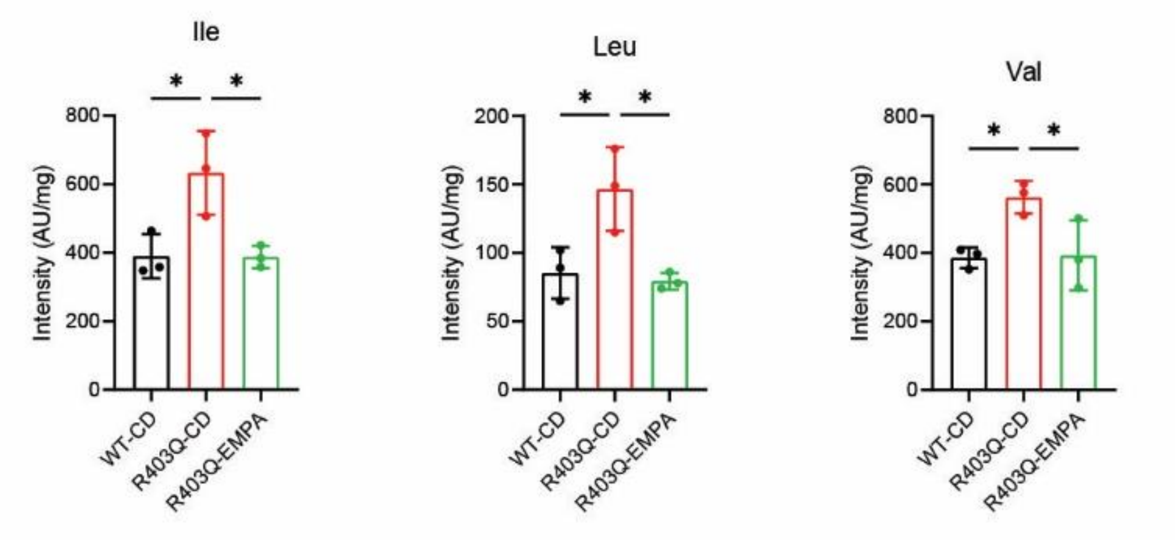


**Figure S14. Cardiac levels of individual BCAAs measured by liquid chromatography-mass spectrometry.** AU, arbitrary unit, Ile, isoleucine, Leu, leucine; Val, valine. Data are means ± SD, n = 3. *P* values were obtained by one-way ANOVA with Bonferroni multiple comparisons tests (adjusted p-value threshold <0.05). **P*<0.05.

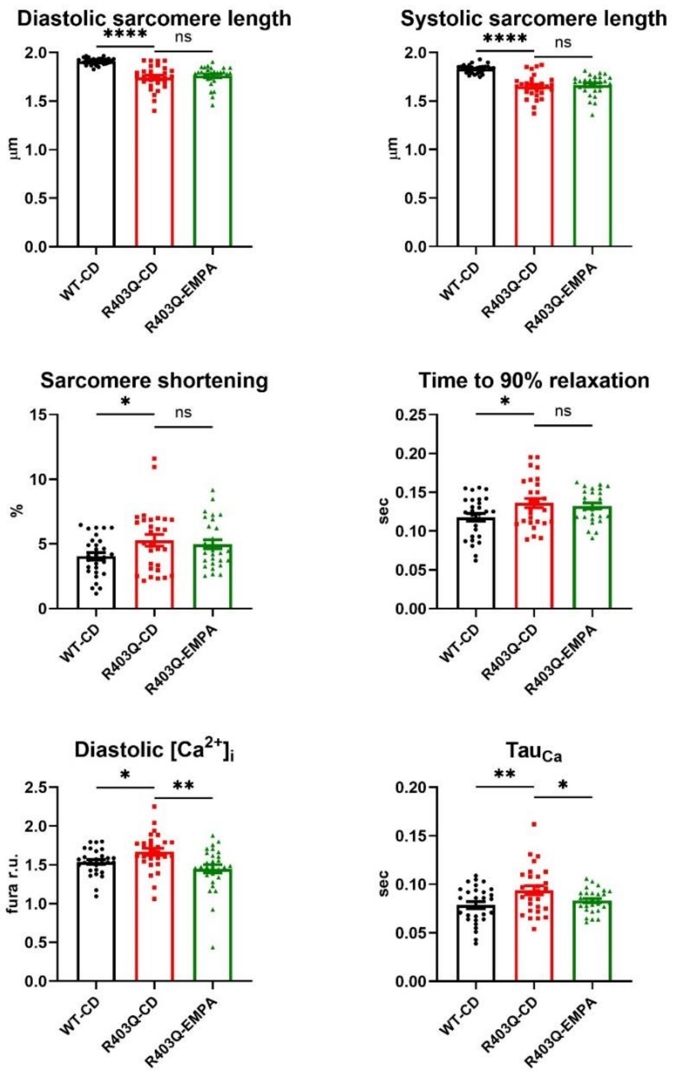


**Figure S15. Sarcomere length, shortening and relaxation and calcium transient in isolated cardiomyocytes after 16-week treatment.** Cardiomyocytes isolated from mice harboring myosin R403Q mutation have shorter sarcomere lengths in systole and diastole, increased sarcomere shortening and impaired relaxation. Furthermore, R403Q mutant cardiomyocytes have increased diastolic intracellular $Ca^{2+}$ and impaired $Ca^{2+}$ diastolic decay as evidenced by increased exponential diastolic $Ca^{2+}$ decay time constant ($Tau_{Ca}$). Empagliflozin improved calcium handling associated with a trend to relaxation normalization. Data shown are mean±SEM. n=28-30 cells isolated from 3 mice per group. *P* values were obtained by one-way ANOVA with Holm-Šídák's multiple comparisons test. $^{*}P<0.05$, $^{**}P<0.01$, $^{****}P<0.0001$; ns, not significant.

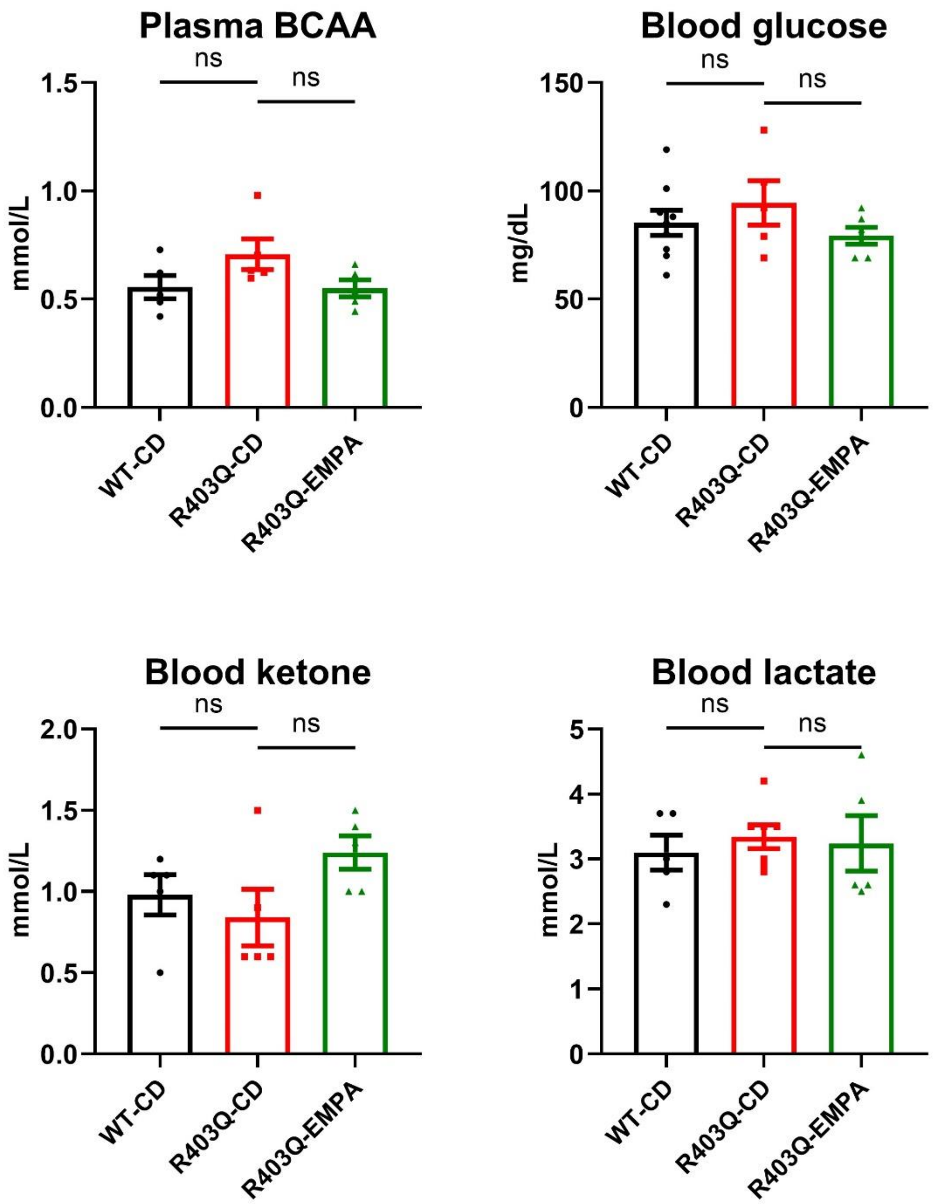


**Figure S16. Metabolic characteristics of mice after 16-week treatment.** Plasma BCAA levels were measured using a colorimetric assay kit (Catalog No. ab83374, Abcam, Cambridge, UK) conforming to the manufacturer instructions. Blood glucose, ketone and lactate levels were measured with particular handheld meters conforming to the manufacturer instructions. Data shown are mean±SEM. n=5-9. *P* values were obtained by one-way ANOVA with Bonferroni multiple comparisons tests (adjusted p-value threshold <0.05). ns, not significant.

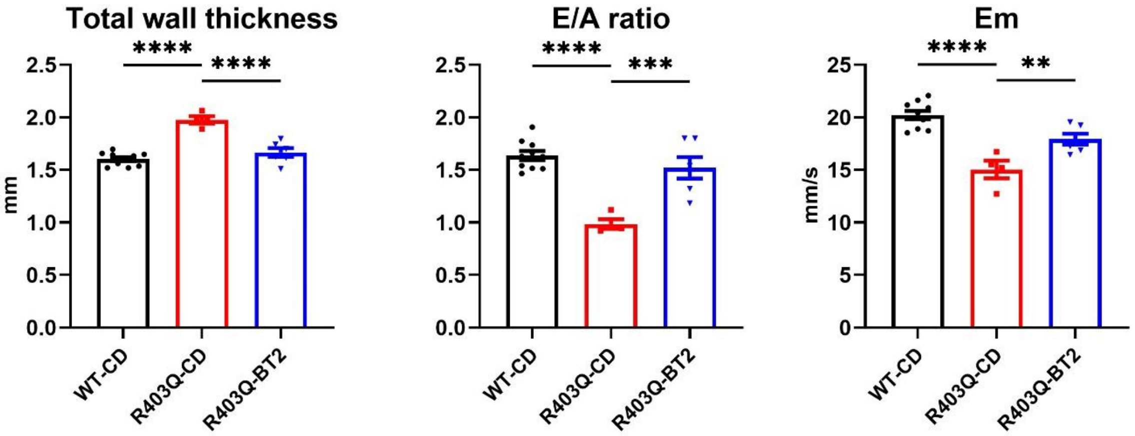


**Figure S17. BT2 improves cardiac hypertrophy and diastolic function in R403Q mutant mice.** A 16-week treatment with 3,6-dichlorobenzo[b]thiophene-2-carboxylic acid (BT2), a potent inhibitor of branched chain ketoacid dehydrogenase kinase (BCKDK) activating BCAA oxidation, improves cardiac hypertrophy and diastolic function in mice harboring myosin R403Q mutation. Data shown are mean±SEM. n=4-10. *P* values were obtained by one-way ANOVA with Bonferroni multiple comparisons tests (adjusted p-value threshold <0.05). ***P*<0.01, ****P*<0.001, *****P*<0.0001.

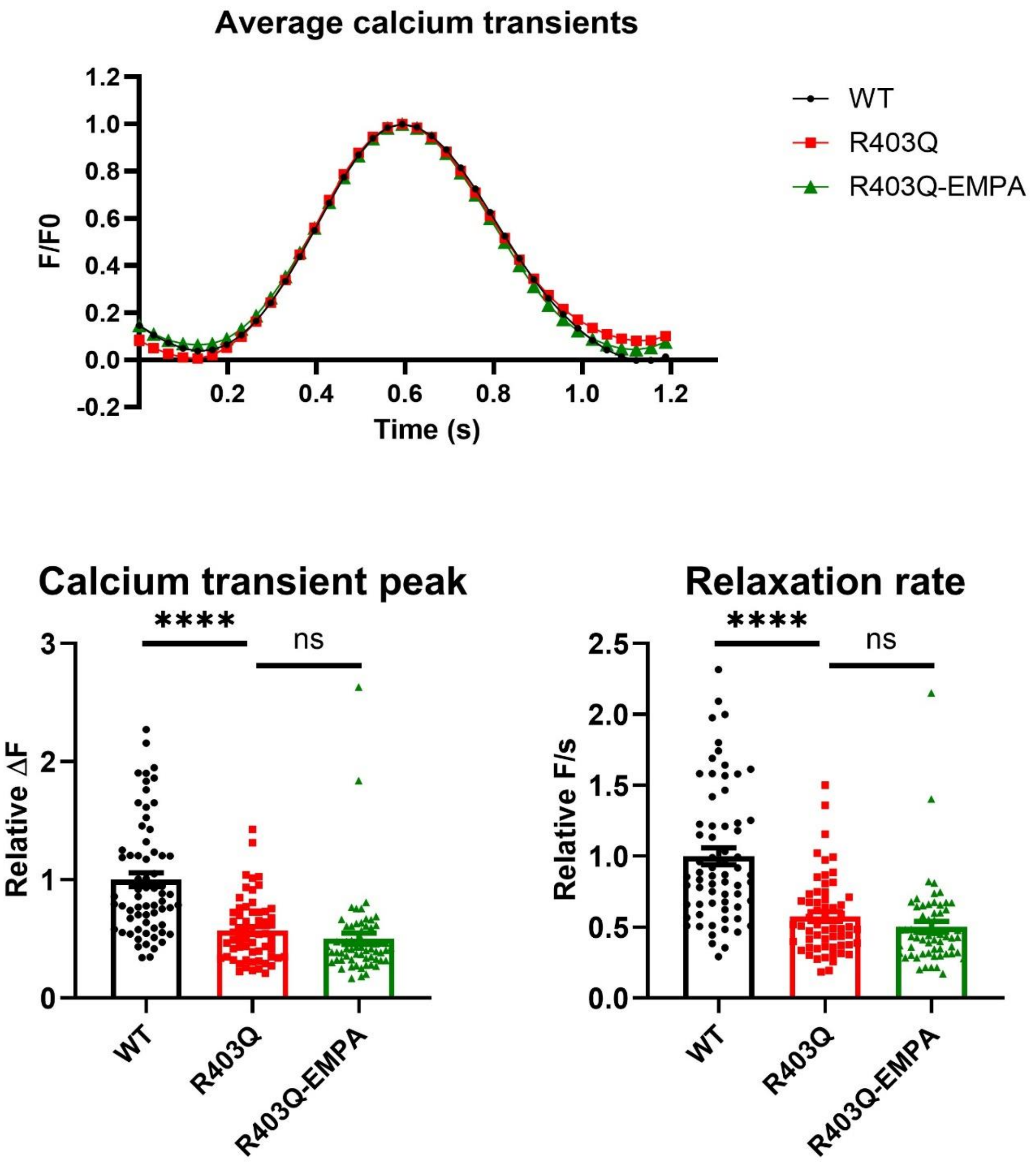


**Figure S18. Calcium transient and relaxation rate of induced pluripotent stem cell-derived cardiomyocytes (iPSC-CM).** iPSC-CMs harboring myosin R403Q mutation present impaired calcium handling and relaxation. Acute treatment with 1 μM EMPA did not have an effect on these parameters. n = 3; n > 60 cells. *P* values were obtained by two-way ANOVA with a mixed-effect model that was corrected for multiple comparisons using a Bonferroni test (adjusted p-value threshold <0.05). *****P*<0.0001; ns, not significant.

## Supplementary Tables

**Table S1. Cardiac RNA sequencing analysis conducted on 12-week-old mice prior to the development of left ventricular hypertrophy (LVH); selected pathways with a FDRq < 0.25 suggestive of pathway enrichment.** R403Q hearts showed the following trends: 1) Upregulation of mTORC1 signaling; 2) Downregulation of fatty acid metabolism; 3) Pathological regulation of energetic metabolism pathways, characterized by the downregulation of mitochondrial electron transport, alongside upregulation of AMP-activated protein kinase (AMPK) activation and ATPase-coupled ion transport; 4) Dysregulation of metabolic signaling pathways, evident from downregulation of insulin signaling and upregulation of glucagon signaling; and 5) Pathological modulation of cardiac muscle contraction and relaxation. Differential expression was assessed using the Wald test implemented in the DESeq2 R package. Sample sizes were n = 5 for WT-CD, n = 7 for R403Q-CD, and n = 4 for R403Q-EMPA. Gene sets were obtained from the Molecular Signatures Database (MSigDB), version 7.5.1. False discovery rate (FDR) corrected p-values (FDRq) and nominal p-values (p) of each gene set are reported. An FDRq < 0.05 was considered significant and an FDRq < 0.25 was considered suggestive of pathway enrichment. Gene sets showing coordinated upregulation are depicted in red; those showing coordinated downregulation are depicted in blue.

| | | | R403Q-CD vs WT-CD | | | R403Q-EMPA vs R403Q-CD | | |
|---|---|---|---|---|---|---|---|---|
| **Keyword** | **Group** | **Gene Set Name** | **NES** | ***p*** | **FDR *q*** | **NES** | ***p*** | **FDR *q*** |
| **Fatty acid** | Hallmark | FATTY ACID METABOLISM | -1.52 | 0.0025 | ***0.1309*** | 1.64 | <0.0001 | ***0.1847*** |
| **mTOR** | Hallmark | MTORC1 SIGNALING | 1.43 | 0.0106 | ***0.1366*** | -1.50 | <0.0001 | ***0.2736*** |
| **ATPase** | C5 GO Molecular Function | ATPASE COUPLED ION TRANSMEMBRANE TRANSPORTER ACTIVITY | 1.48 | 0.0545 | ***0.1019*** | -1.57 | 0.0289 | ***0.2124*** |
| **Insulin** | C5 GO Biological Process | RESPONSE TO INSULIN | -1.55 | <0.0001 | ***0.1203*** | 1.60 | <0.0001 | ***0.2006*** |
| | C2 Reactome pathway | INSULIN RECEPTOR SIGNALLING CASCADE | -1.52 | 0.0197 | ***0.1327*** | 1.59 | 0.0140 | ***0.2039*** |
| | C5 GO Biological Process | CELLULAR RESPONSE TO INSULIN STIMULUS | -1.52 | <0.0001 | ***0.1331*** | 1.49 | 0.0025 | ***0.2364*** |
| **Glucagon** | C2 Reactome pathway | SYNTHESIS SECRETION AND INACTIVATION OF GLUCAGON LIKE PEPTIDE 1 GLP 1 | 1.58 | 0.0152 | ***0.0582*** | -1.59 | 0.0220 | ***0.1999*** |
| **AMPK** | C2 Reactome pathway | ACTIVATION OF AMPK DOWNSTREAM OF NMDARS | 1.48 | 0.0509 | ***0.1016*** | -1.62 | 0.0185 | ***0.1749*** |
| **Mitochondria** | C5 GO Biological Process | MITOCHONDRIAL ELECTRON TRANSPORT NADH TO UBIQUINONE | -1.40 | 0.0625 | ***0.1952*** | 1.47 | 0.0285 | ***0.2490*** |
| | C5 GO Biological Process | MITOCHONDRIAL DNA METABOLIC PROCESS | -1.49 | 0.0516 | ***0.1429*** | 1.54 | 0.0409 | ***0.2201*** |
| **Misc.** | C2 WikiPathways pathway | MYOMETRIAL RELAXATION AND CONTRACTION PATHWAYS | 1.30 | 0.0417 | ***0.2475*** | -1.59 | 0.0050 | ***0.2031*** |

**Table S2. Top 20 most up- or down-regulated gene sets by EMPA in R403Q hearts.** Hearts of 12-week-old animals before LV hypertrophy and after 2 weeks of EMPA treatment were used. Differential expression was assessed using the Wald test implemented in the DESeq2 R package. n = 5 (WT-CD), 7 (R403Q-CD), and 4 (R403Q-EMPA). Gene sets obtained from the Molecular Signatures Database (MSigDB), version 7.5.1. FDRq, Benjamini-Hochberg false discovery rate-corrected p-value. NES, Normalized Enrichment Score. p, nominal p value. FDRq <0.05 was considered significant. Gene sets coordinately upregulated are depicted in red; Gene sets coordinately downregulated are depicted in blue.

| **TOP 20 DOWNREGULATED PATHWAYS BY EMPA IN R403Q HEARTS** | | **R403Q-EMPA vs R403Q-CD** | | |
|---|---|---|---|---|
| **Group** | **Gene Set Name** | **NES** | ***p*** | **FDR *q*** |
| C2 Reactome pathway | COLLAGEN CHAIN TRIMERIZATION | -2.36 | <0.0001 | ***<0.0001*** |
| C2 Reactome pathway | COLLAGEN BIOSYNTHESIS AND MODIFYING ENZYMES | -2.34 | <0.0001 | ***<0.0001*** |
| C5 GO Molecular Function | EXTRACELLULAR MATRIX STRUCTURAL CONSTITUENT CONFERRING TENSILE STRENGTH | -2.29 | <0.0001 | ***0.0003*** |
| C5 GO Cellular Component | COLLAGEN TRIMER | -2.28 | <0.0001 | ***0.0002*** |
| C2 Reactome pathway | COLLAGEN FORMATION | -2.25 | <0.0001 | ***0.0014*** |
| C2 Reactome pathway | SMOOTH MUSCLE CONTRACTION | -2.24 | <0.0001 | ***0.0016*** |
| Hallmark | EPITHELIAL MESENCHYMAL TRANSITION | -2.17 | <0.0001 | ***0.0041*** |
| C2 WikiPathways pathway | PATHOGENIC ESCHERICHIA COLI INFECTION | -2.17 | <0.0001 | ***0.0036*** |
| C2 Reactome pathway | APC C MEDIATED DEGRADATION OF CELL CYCLE PROTEINS | -2.15 | <0.0001 | ***0.0050*** |
| C2 Reactome pathway | THE ROLE OF GTSE1 IN G2 M PROGRESSION AFTER G2 CHECKPOINT | -2.14 | <0.0001 | ***0.0061*** |
| C2 KEGG pathway | PATHOGENIC ESCHERICHIA COLI INFECTION | -2.13 | <0.0001 | ***0.0057*** |
| C2 KEGG pathway | PROTEASOME | -2.11 | <0.0001 | ***0.0081*** |
| C2 Reactome pathway | GOLGI TO ER RETROGRADE TRANSPORT | -2.11 | <0.0001 | ***0.0077*** |
| C5 GO Molecular Function | EXTRACELLULAR MATRIX STRUCTURAL CONSTITUENT | -2.10 | <0.0001 | ***0.0075*** |
| C5 GO Cellular Component | COMPLEX OF COLLAGEN TRIMERS | -2.10 | <0.0001 | ***0.0072*** |
| Hallmark | INTERFERON ALPHA RESPONSE | -2.10 | <0.0001 | ***0.0070*** |
| C5 GO Cellular Component | PROTEASOME COMPLEX | -2.07 | <0.0001 | ***0.0104*** |
| C2 Reactome pathway | METABOLISM OF POLYAMINES | -2.06 | <0.0001 | ***0.0109*** |
| C2 PID pathway | AURORA B PATHWAY | -2.05 | <0.0001 | ***0.0124*** |
| C2 Reactome pathway | SCF SKP2 MEDIATED DEGRADATION OF P27 P21 | -2.05 | <0.0001 | ***0.0127*** |

| **TOP 20 UPREGULATED PATHWAYS BY EMPA IN R403Q HEARTS** | | **R403Q-EMPA vs R403Q-CD** | | |
|---|---|---|---|---|
| **Group** | **Gene Set Name** | **NES** | ***p*** | **FDR *q*** |
| C2 WikiPathways pathway | FATTY ACID BETAOXIDATION | 2.38 | <0.0001 | ***0.0023*** |
| C5 GO Biological Process | FATTY ACID BETA OXIDATION | 2.30 | <0.0001 | ***0.0046*** |
| C5 GO Biological Process | CELLULAR RESPONSE TO STEROL DEPLETION | 2.25 | <0.0001 | ***0.0096*** |
| C2 Reactome pathway | NR1H3 NR1H2 REGULATE GENE EXPRESSION LINKED TO CHOLESTEROL TRANSPORT AND EFFLUX | 2.21 | <0.0001 | ***0.0142*** |
| C5 GO Biological Process | NEGATIVE REGULATION OF FATTY ACID METABOLIC PROCESS | 2.15 | <0.0001 | ***0.0245*** |
| C2 WikiPathways pathway | MITOCHONDRIAL LONG CHAIN FATTY ACID BETAOXIDATION | 2.13 | <0.0001 | ***0.0282*** |
| C5 GO Biological Process | FATTY ACID CATABOLIC PROCESS | 2.12 | <0.0001 | ***0.0268*** |
| C2 KEGG pathway | PPAR SIGNALING PATHWAY | 2.11 | <0.0001 | ***0.0297*** |
| C2 Reactome pathway | NR1H2 AND NR1H3 MEDIATED SIGNALING | 2.10 | <0.0001 | ***0.0290*** |
| C5 GO Biological Process | REGULATION OF FATTY ACID BETA OXIDATION | 2.10 | <0.0001 | ***0.0268*** |
| C2 Reactome pathway | MITOCHONDRIAL FATTY ACID BETA OXIDATION | 2.09 | <0.0001 | ***0.0292*** |
| C3 TF motifs | WYAAANNRNNNGCG UNKNOWN | 2.08 | <0.0001 | ***0.0292*** |
| C5 GO Biological Process | REGULATION OF TRIGLYCERIDE METABOLIC PROCESS | 2.08 | <0.0001 | ***0.0278*** |
| C2 WikiPathways pathway | PPAR SIGNALING PATHWAY | 2.07 | <0.0001 | ***0.0286*** |
| C5 GO Cellular Component | MICROBODY LUMEN | 2.06 | <0.0001 | ***0.0302*** |
| C2 WikiPathways pathway | PATHWAYS AFFECTED IN ADENOID CYSTIC CARCINOMA | 2.04 | <0.0001 | ***0.0388*** |
| C2 Reactome pathway | REGULATION OF LIPID METABOLISM BY PPARALPHA | 2.03 | <0.0001 | ***0.0404*** |
| C5 GO Biological Process | FATTY ACID TRANSMEMBRANE TRANSPORT | 2.03 | 0.0022 | ***0.0411*** |
| C5 GO Biological Process | REGULATION OF TRIGLYCERIDE BIOSYNTHETIC PROCESS | 2.02 | 0.0022 | ***0.0421*** |
| C3 microRNA motifs | MIR1233 3P | 2.02 | <0.0001 | ***0.0410*** |

**The biological significance of the pathways in Table S2**

**Down-regulated gene sets by EMPA in R403Q hearts:**

One of the phenotypic hallmarks of HCM is cardiac fibrosis.[1,42] In our analysis, gene sets associated with **collagen metabolism** were coordinately upregulated in R403Q hearts and **downregulated by EMPA**, as shown in Table 1. Notably, EMPA's effect on collagen-related pathways was among the most significantly regulated, with 7 of the 20 most downregulated gene sets linked to collagen or extracellular matrix remodeling. These included: Collagen chain trimerization, Collagen biosynthesis and modifying enzymes, Extracellular matrix structural constituent conferring tensile strength, Collagen trimer, Collagen formation, Extracellular matrix structural constituent, and Complex of collagen trimers. These findings reinforce previous evidence showing that SGLT2 inhibitors alleviate cardiac fibrosis in experimental models of various cardiovascular pathologies, including heart failure with reduced ejection fraction after myocardial infarction, heart failure with preserved ejection fraction, or diabetic cardiomyopathy.[8,43,44] Moreover, our histology analysis further supports the transcriptomic data showing attenuation of the profound cardiac fibrosis in R403Q hearts by EMPA (Figure 6F).

Another phenotypic hallmark of HCM is cardiomyocyte hypertrophy.[1,42] Notably, 8 out of 20 most down-regulated gene sets by EMPA were associated with cell growth and proliferation encompassing key biological processes such as DNA replication, RNA transcription, protein turnover, post-translational modifications, and apoptosis. These included APC/C-mediated degradation of cell cycle proteins, The role of GTSE1 in G2/M progression after G2 checkpoint, Proteasome, Golgi to ER retrograde transport, Proteasome complex, Metabolism of polyamines, Aurora B pathway, and SCF(Skp2)-mediated degradation of p27/p21. These findings are consistent with prior studies demonstrating the **anti-remodeling effects of SGLT2 inhibitors** across various cardiovascular conditions.[8,45,46] Moreover, our histological analysis corroborates these transcriptomic changes, revealing a significant

reduction in cardiomyocyte cross-sectional area in R403Q hearts following EMPA treatment (Figure 6E).

There is also increasing evidence of low-grade systemic and cardiac inflammation in HCM.[47,48] Interestingly, SGLT2 inhibitors were shown to exert **anti-inflammatory properties**.[49] Indeed, 3 out of 20 most down-regulated gene sets by EMPA were associated with inflammatory response, including Pathogenic Escherichia coli infection in both WikiPatways and KEGG databases, and Interferon alpha response.

Finally, the relevance of the remaining two most downregulated gene sets - Smooth muscle cell contraction and Epithelial-mesenchymal transition - is unclear and warrants further investigation.

**Up-regulated gene sets by EMPA in R403Q hearts:**

Notably, 16 out of the 20 most **up-regulated** gene sets by EMPA were associated with lipid metabolism including **fatty acid, triglyceride, and cholesterol metabolism and their associated signaling pathways**. These included: Fatty acid oxidation (WikiPathways and GOBP databases), Cellular response to sterol depletion, NR1H3 and NR1H2 regulate gene expression linked to cholesterol transport and efflux, Negative regulation of fatty acid metabolic process, Mitochondrial long-chain fatty acid betaoxidation, Fatty acid catabolic process, PPAR signaling pathway, NR1H2 and NR1H3 mediated signaling, Regulation of fatty acid betaoxidation, Mitochondrial fatty acid betaoxidation, Regulation of triglyceride metabolic process, PPAR signaling pathway, Regulation of lipid metabolism by PPARalpha, Fatty acid transmembrane transport, and Regulation of triglyceride biosynthetic process. These findings align with our functional metabolic data (Table 1 and Figure 3E) demonstrating increased Fatty acid oxidation in R403Q hearts following EMPA treatment, as measured by substrate oxidation fluxes). This supports the notion that **enhancing mitochondrial oxidative capacity is a key mechanism of EMPA's cardioprotective effects in HCM**. Additionally, these results are

consistent with prior studies demonstrating similar metabolic benefits of SGLT2 inhibitors in other cardiac pathologies.[8,50]

The functional relevance of the remaining four gene sets up-regulated by EMPA (WYAAANNRNNNGCG motif, Microbody lumen, Pathways affected in adenoid cystic carcinoma, and miR-1233-3p motif) remains unclear and may warrant further investigation.

**Table S3. Subset of notable up- and down regulated proteome and phospho-proteome pathways.** Cardiac proteomics/phosphoproteomics. FDR, Benjamini-Hochberg false discovery rate-corrected p-value. NES, Normalized Enrichment Score. p, nominal p value. FDR <0.05 in the R40Q-CD vs wild type-CD was considered significant. Protein/phosphoprotein sets coordinately upregulated are depicted in red; Protein/phosphoprotein sets coordinately downregulated are depicted in blue.

| PROTEOMICS | | | R403Q-CD vs WT-CD | | | R403Q-EMPA vs R403Q-CD | | |
|---|---|---|---|---|---|---|---|---|
| **Keyword** | **Group** | **Term** | **NES** | **p** | **FDR** | **NES** | **p** | **FDR** |
| Fatty Acid | GO Biological Process | FATTY ACID OXIDATION | -1.88 | 0.0003 | **0.0056** | 1.83 | 0.0008 | **0.0288** |
| | GO Biological Process | FATTY ACID CATABOLIC PROCESS | -1.98 | 0.0002 | **0.0056** | 1.97 | 0.0002 | **0.0189** |
| | Reactome Pathway | MITOCHONDRIAL FATTY ACID BETA-OXIDATION | -1.94 | 0.0003 | **0.0056** | 1.83 | 0.0022 | **0.0488** |
| | GO Biological Process | FATTY ACID BETA-OXIDATION | -1.99 | 0.0003 | **0.0056** | 1.86 | 0.0006 | **0.0238** |
| | Reactome Pathway | FATTY ACID METABOLISM | -1.64 | 0.0022 | **0.0169** | 1.83 | 0.0002 | **0.0189** |
| Pyruvate | Reactome Pathway | PYRUVATE METABOLISM AND CITRIC ACID (TCA) CYCLE | -1.83 | 0.0006 | **0.0061** | 1.65 | 0.0064 | **0.0896** |
| The citric acid cycle and oxidative phosphorylation | Reactome Pathway | THE CITRIC ACID (TCA) CYCLE AND RESPIRATORY ELECTRON TRANSPORT | -2.55 | 0.0001 | **0.0056** | 1.92 | 0.0001 | **0.0189** |
| | WikiPathways | OXIDATIVE PHOSPHORYLATION | -2.37 | 0.0002 | **0.0056** | 1.72 | 0.0046 | **0.0749** |
| | GO Biological Process | PROTON MOTIVE FORCE-DRIVEN ATP SYNTHESIS | -2.49 | 0.0002 | **0.0056** | 1.36 | 0.0755 | **0.3723** |
| | GO Biological Process | PROTON MOTIVE FORCE-DRIVEN MITOCHONDRIAL ATP SYNTHESIS | -2.52 | 0.0002 | **0.0056** | 1.36 | 0.0755 | **0.3723** |
| Collagen | Reactome Pathway | COLLAGEN FORMATION | 1.90 | 0.0008 | **0.0074** | -1.47 | 0.0356 | **0.2455** |
| | GO Biological Process | REGULATION OF COLLAGEN METABOLIC PROCESS | 2.01 | 0.0005 | **0.0057** | -1.71 | 0.0105 | **0.1257** |
| | GO Biological Process | COLLAGEN FIBRIL ORGANIZATION | 2.06 | 0.0003 | **0.0056** | -1.62 | 0.0184 | **0.1718** |
| | Reactome Pathway | ASSEMBLY OF COLLAGEN FIBRILS AND OTHER MULTIMERIC STRUCTURES | 2.23 | 0.0003 | **0.0056** | -1.95 | 0.0007 | **0.0269** |
| | Reactome Pathway | COLLAGEN CHAIN TRIMERIZATION | 2.39 | 0.0002 | **0.0056** | -2.09 | 0.0002 | **0.0189** |
| IGF | Reactome Pathway | REGULATION OF IGF ACTIVITY BY IGFBP | 2.16 | 0.0003 | **0.0056** | -1.51 | 0.0175 | **0.1676** |
| mTOR | WikiPathways | FOCAL ADHESION: PI3K-AKT-MTOR SIGNALING PATHWAY | 1.83 | 0.0004 | **0.0056** | -1.79 | 0.0003 | **0.0189** |

| PHOSPHO-PROTEOMICS | | | R403Q-CD vs WT-CD | | | R403Q-EMPA vs R403Q-CD | | |
|---|---|---|---|---|---|---|---|---|
| **Keyword** | **Group** | **Term** | **NES** | **p** | **FDR** | **NES** | **p** | **FDR** |
| Glucose | GO Biological Process | GLUCOSE METABOLIC PROCESS | -1.50 | 0.0450 | **0.2772** | 1.86 | 0.0015 | **0.0505** |
| The citric acid cycle | Reactome Pathway | THE CITRIC ACID (TCA) CYCLE AND RESPIRATORY ELECTRON TRANSPORT | -2.11 | 0.0002 | **0.0119** | 1.98 | 0.0005 | **0.0294** |
| Oxidative phosphorylation and ATP | GO Biological Process | OXIDATIVE PHOSPHORYLATION | -2.01 | 0.0004 | **0.0207** | 1.88 | 0.0026 | **0.0808** |
| | GO Biological Process | ATP METABOLIC PROCESS | -1.87 | 0.0013 | **0.0436** | 2.01 | 0.0003 | **0.0257** |
| Heart contraction | GO Biological Process | REGULATION OF THE FORCE OF HEART CONTRACTION | -2.09 | 0.0004 | **0.0207** | 1.70 | 0.0136 | **0.2307** |

**Table S4. Heart and body weights after 16-week EMPA treatment.** Data shown are mean±SEM. n=6-7. *P* values were obtained by one-way ANOVA with Bonferroni multiple comparisons tests (adjusted p-value threshold <0.05). *$P$<0.05 vs. WT-CD; #$P$<0.05 vs. R403Q-CD.

| | WT-CD | R403Q-CD | R403Q-EMPA |
|---|---|---|---|
| **Body weight (BW)** (g) | 27.38 ± 1.67 | 29.83 ± 1.45 | 29.73 ± 1.99 |
| **Heart weight** (mg) | 123.13 ± 6.21 | 136.46 ± 3.17 | 122.18 ± 2.21 |
| **LV weight** (mg) | 80.41 ± 3.44 | 93.96 ± 2.33* | 82.78 ± 2.72# |
| **Heart weight/BW** (mg/g) | 4.52 ± 0.10 | 4.67 ± 0.35 | 4.19 ± 0.27 |
| **LV weight/BW** (mg/g) | 2.97 ± 0.11 | 3.21 ± 0.22 | 2.82 ± 0.14 |

**Table S5. Mean, SEM and N for all experiments and including the WT-EMPA group.** Of note, RNA sequencing datasets (Table 1) have been deposited in Gene Expression Omnibus (GEO Series ID: GSE270093) and proteomics and phosphoproteomics data (Figure 2) have been deposited to the ProteomeXchange Consortium via the PRIDE partner repository with the dataset identifier PXD053046 and 10.6019/PXD053046.

| Figure | Panel | WT-CD | | WT-EMPA | | R403Q-CD | | R403Q-EMPA | |
|---|---|---|---|---|---|---|---|---|---|
| | | mean ± SEM | n | mean ± SEM | n | mean ± SEM | n | mean ± SEM | n |
| Figure 3 | A | 0.59 ± 0.19 | 5 | 0.38 ± 0.11 | 3 | 1.58 ± 0.31 | 5 | 0.55 ± 0.10 | 4 |
| | B | 0.35 ± 0.4 | 3 | 0.11 ± 0.03 | 4 | 0.98 ± 0.05 | 4 | 0.34 ± 0.01 | 3 |
| | C | 0.15 ± 0.09 | 3 | 0.01 ± 0.05 | 4 | 0.55 ± 0.10 | 4 | 0.04 ± 0.03 | 3 |
| | D | 0.75 ± 0.25 | 3 | 0.28 ± 0.02 | 3 | 1.89 ± 0.16 | 4 | 0.87 ± 0.22 | 3 |
| | E | 0.43 ± 0.01 | 3 | 0.37 ± 0.01 | 4 | 0.16 ± 0.02 | 4 | 0.44 ± 0.01 | 3 |
| | F | 100.00 ± 6.58 | 4 | n/a | n/a | 143.00 ± 13.18 | 5 | 96.03 ± 6.59 | 4 |
| Figure 4 | A at LW | 41.01 ± 1.13 | 7 | 56.18 ± 5.99 | 6 | 56.25 ± 3.02 | 9 | 55.35 ± 3.54 | 6 |
| | A at HW | 76.80 ± 4.24 | 7 | 109.61 ± 14.51 | 4 | 59.00 ± 4.05 | 9 | 93.20 ± 7.16 | 6 |
| | B at LW | 91.14 ± 2.51 | 7 | 135.50 ± 12.96 | 6 | 125.00 ± 6.70 | 9 | 123.00 ± 7.87 | 6 |
| | B at HW | 128.00 ± 7.06 | 7 | 202.80 ± 22.56 | 5 | 98.33 ± 6.74 | 9 | 153.00 ± 13.65 | 6 |
| | C at LW | 8.57 ± 0.30 | 7 | 9.00 ± 0.68 | 6 | 10.11 ± 0.66 | 9 | 9.00 ± 0.37 | 6 |
| | C at HW | 11.57 ± 0.61 | 7 | 11.67 ± 2.35 | 6 | 21.33 ± 2.26 | 9 | 11.83 ± 1.28 | 6 |
| | D at LW | 53.55 ± 16.92 | 7 | 75.51 ± 25.31 | 6 | 83.85 ± 8.53 | 9 | 120.46 ± 23.86 | 6 |
| | D at HW | 110.47 ± 27.41 | 7 | 82.30 ± 42.20 | 4 | 149.43 ± 15.65 | 9 | 82.21 ± 14.21 | 6 |
| | E at LW | 38.19 ± 1.67 | 7 | 46.53 ± 5.91 | 6 | 75.14 ± 2.97 | 9 | 54.95 ± 3.45 | 6 |
| | E at HW | 72.69 ± 7.12 | 7 | 90.18 ± 8.87 | 4 | 81.14 ± 4.15 | 9 | 95.57 ± 8.21 | 6 |
| | F at LW | 57.30 ± 0.74 | 7 | 57.05 ± 0.74 | 6 | 54.16 ± 0.25 | 9 | 54.22 ± 0.28 | 6 |
| | F at HW | 53.97 ± 0.26 | 7 | 55.83 ± 0.94 | 4 | 52.00 ± 0.31 | 9 | 53.77 ± 0.40 | 6 |
| | G | n/a | n/a | n/a | n/a | n/a | n/a | n/a | n/a |
| | H at LW | 936.29 ± 51.79 | 7 | 773.17 ± 109.35 | 6 | 1356.00 ± 62.58 | 9 | 1018.33 ± 105.91 | 6 |
| | H at HW | 1061.57 ± 80.60 | 7 | 827.50 ± 150.55 | 4 | 1390.33 ± 43.64 | 9 | 1068.67 ± 134.80 | 6 |
| Figure 5 | A | 1.58 ± 0.02 | 6 | 1.58 ± 0.03 | 6 | 1.95 ± 0.05 | 6 | 1.72 ± 0.05 | 6 |
| | B | 28.18 ± 1.00 | 6 | 30.73 ± 2.90 | 6 | 40.46 ± 3.46 | 6 | 41.38 ± 3.83 | 6 |
| | C | 1.53 ± 0.06 | 6 | 1.65 ± 0.09 | 6 | 0.94 ± 0.05 | 6 | 1.29 ± 0.05 | 6 |
| | D | 23.92 ± 0.55 | 6 | 23.52 ± 0.54 | 6 | 15.11 ± 0.45 | 6 | 21.29 ± 0.56 | 6 |
| | E | 329.04 ± 12.29 | 8 | 252.54 ± 15.24 | 5 | 546.56 ± 30.64 | 8 | 318.24 ± 15.11 | 8 |
| | F | 0.58 ± 0.04 | 8 | 0.64 ± 0.05 | 5 | 2.13 ± 0.20 | 8 | 1.07 ± 0.04 | 8 |
| Figure 6 | A | 1.00 ± 0.21 | 5 | 1.07 ± 0.18 | 4 | 2.03 ± 0.15 | 5 | 1.15 ± 0.12 | 5 |
| | B | 1.00 ± 0.68 | 5 | n/a | n/a | 6.32 ± 0.65 | 5 | 0.51 ± 0.17 | 5 |
| | C | 10.50 ± 0.25 | 5 | 9.26 ± 1.18 | 4 | 10.96 ± 1.10 | 6 | 7.52 ± 0.72 | 4 |

## References


1. Geisterfer-Lowrance AAT, Christe M, Conner DA, Ingwall JS, Schoen FJ, Seidman CE, et al. A mouse model of familial hypertrophic cardiomyopathy. *Science* 1996;**272**:731–734. https://doi.org/10.1126/SCIENCE.272.5262.731

2. Green EM, Wakimoto H, Anderson RL, Evanchik MJ, Gorham JM, Harrison BC, et al. A small-molecule inhibitor of sarcomere contractility suppresses hypertrophic cardiomyopathy in mice. *Science* 2016;**351**:617–621. https://doi.org/10.1126/SCIENCE.AAD3456

3. Reichart D, Newby GA, Wakimoto H, Lun M, Gorham JM, Curran JJ, et al. Efficient in vivo genome editing prevents hypertrophic cardiomyopathy in mice. *Nat Med* 2023;**29**:412–421. https://doi.org/10.1038/S41591-022-02190-7

4. He H, Hoyer K, Tao H, Rice R, Jimenez J, Tardiff JC, et al. Myosin-driven rescue of contractile reserve and energetics in mouse hearts bearing familial hypertrophic cardiomyopathy-associated mutant troponin T is mutation-specific. *J Physiol* 2012;**590**:5371–5388. https://doi.org/10.1113/JPHYSIOL.2012.234252

5. He H, Javadpour MM, Latif F, Tardiff JC, Ingwall JS. R-92L and R-92W mutations in cardiac troponin T lead to distinct energetic phenotypes in intact mouse hearts. *Biophys J* 2007;**93**:1834–1844. https://doi.org/10.1529/BIOPHYSJ.107.107557

6. Ertz-Berger BR, He H, Dowell C, Factor SM, Haim TE, Nunez S, et al. Changes in the chemical and dynamic properties of cardiac troponin T cause discrete cardiomyopathies in transgenic mice. *Proc Natl Acad Sci U S A* 2005;**102**:18219–18224. https://doi.org/10.1073/PNAS.0509181102

7. Murashige D, Jung JW, Neinast MD, Levin MG, Chu Q, Lambert JP, et al. Extra-cardiac BCAA catabolism lowers blood pressure and protects from heart failure. *Cell Metab* 2022;**34**:1749-1764.e7. https://doi.org/10.1016/J.CMET.2022.09.008

8. Croteau D, Luptak I, Chambers JM, Hobai I, Panagia M, Pimentel DR, et al. Effects of Sodium-Glucose Linked Transporter 2 Inhibition With Ertugliflozin on Mitochondrial Function, Energetics, and Metabolic Gene Expression in the Presence and Absence of Diabetes Mellitus in Mice. *J Am Heart Assoc* 2021;**10**:e019995. https://doi.org/10.1161/JAHA.120.019995

9. Qin F, Siwik DA, Luptak I, Hou X, Wang L, Higuchi A, et al. The polyphenols resveratrol and S17834 prevent the structural and functional sequelae of diet-induced metabolic heart disease in mice. *Circulation* 2012;**125**:1757–1764. https://doi.org/10.1161/CIRCULATIONAHA.111.067801

10. Luptak I, Qin F, Sverdlov AL, Pimentel DR, Panagia M, Croteau D, et al. Energetic Dysfunction Is Mediated by Mitochondrial Reactive Oxygen Species and Precedes Structural Remodeling in Metabolic Heart Disease. *Antioxid Redox Signal* 2019;**31**:539–549. https://doi.org/10.1089/ars.2018.7707

11. Croteau D, Baka T, Young S, He H, Chambers JM, Qin F, et al. SGLT2 inhibitor ertugliflozin decreases elevated intracellular sodium, and improves energetics and contractile function in diabetic cardiomyopathy. *Biomed Pharmacother* 2023;**160**:114310. https://doi.org/10.1016/J.BIOPHA.2023.114310

12. Kammermeier H. High energy phosphate of the myocardium: concentration versus free energy change. *Basic Res Cardiol* 1987;**82 Suppl 2**:31–36. https://doi.org/10.1007/978-3-662-11289-2_3

13. Luptak I, Sverdlov AL, Panagia M, Qin F, Pimentel DR, Croteau D, et al. Decreased ATP production and myocardial contractile reserve in metabolic heart disease. *J Mol Cell Cardiol* 2018;**116**:106–114. https://doi.org/10.1016/j.yjmcc.2018.01.017

14. He H, Baka T, Balschi J, Motani AS, Nguyen KK, Liu Q, et al. Novel Small-Molecule Troponin Activator Increases Cardiac Contractile Function Without Negative Impact on Energetics. *Circ Heart Fail* 2022;**15**:e009195. https://doi.org/10.1161/CIRCHEARTFAILURE.121.009195

15. Panagia M, He H, Baka T, Pimentel DR, Croteau D, Bachschmid MM, et al. Increasing mitochondrial ATP synthesis with butyrate normalizes ADP and contractile function in metabolic heart disease. *NMR Biomed* 2020;**33**. https://doi.org/10.1002/NBM.4258

16. Luptak I, Shen M, He H, Hirshman MF, Musi N, Goodyear LJ, et al. Aberrant activation of AMP-activated protein kinase remodels metabolic network in favor of cardiac glycogen storage. *J Clin Investig* 2007;**117**:1432–1439. https://doi.org/10.1172/JCI30658.1432

17. Luptak I, Yan J, Cui L, Jain M, Liao R, Tian R. Long-term effects of increased glucose entry on mouse hearts during normal aging and ischemic stress. *Circulation* 2007;**116**:901–909. https://doi.org/10.1161/CIRCULATIONAHA.107.691253

18. Luptak I, Balschi JA, Xing Y, Leone TC, Kelly DP, Tian R. Decreased contractile and metabolic reserve in peroxisome proliferator-activated receptor-α-null hearts can be rescued by increasing glucose transport and utilization. *Circulation* 2005;**112**:2339–2346. https://doi.org/10.1161/CIRCULATIONAHA.105.534594

19. Malloy CR, Sherry AD, Jeffrey H. Evaluation of Carbon Flux and Substrate Selection through Alternate Pathways Involving the Citric Acid Cycle of the Heart by 13C NMR Spectroscopy. *J Biol Chem* 1988;**263**:6964–6971. https://doi.org/10.1016/S0021-9258(18)68590-4

20. Liao R, Jain M, Cui L, D'Agostino J, Aiello F, Luptak I, et al. Cardiac-specific overexpression of GLUT1 prevents the development of heart failure attributable to pressure overload in mice. *Circulation* 2002;**106**:2125–2131. https://doi.org/10.1161/01.CIR.0000034049.61181.F3

21. Clarke K, Anderson RE, Nédélec J -F, Foster DO, Ally A. Intracellular and extracellular spaces and the direct quantification of molar intracellular concentrations of phosphorus metabolites in the isolated rat heart using 31P NMR spectroscopy and phosphonate markers. *Magn Reson Med* 1994;**32**:181–188. https://doi.org/10.1002/MRM.1910320206

22. Jansen MA, Shen H, Zhang L, Wolkowicz PE, Balschi JA. Energy requirements for the Na+ gradient in the oxygenated isolated heart: effect of changing the free energy of ATP hydrolysis. *Am J Physiol Heart Circ Physiol* 2003;**285**. https://doi.org/10.1152/AJPHEART.00534.2003

23. Miller EJ, Calamaras T, Elezaby A, Sverdlov A, Qin F, Luptak I, et al. Partial Liver Kinase B1 (LKB1) Deficiency Promotes Diastolic Dysfunction, De Novo Systolic Dysfunction, Apoptosis, and Mitochondrial Dysfunction With Dietary Metabolic Challenge. *J Am Heart Assoc* 2015;**5**:e002277. https://doi.org/10.1161/JAHA.115.002277

24. Moore J, Ewoldt J, Venturini G, Pereira AC, Padilha K, Lawton M, et al. Multi-Omics Profiling of Hypertrophic Cardiomyopathy Reveals Altered Mechanisms in Mitochondrial Dynamics and Excitation–Contraction Coupling. *Int J Mol Sci* 2023;**24**:4724. https://doi.org/10.3390/IJMS24054724/S1

25. Hobai IA, Buys ES, Morse JC, Edgecomb J, Weiss EH, Armoundas AA, et al. SERCA Cys674 sulphonylation and inhibition of L-type Ca2+ influx contribute to cardiac dysfunction in endotoxemic mice, independent of cGMP synthesis. *Am J Physiol Heart Circ Physiol* 2013;**305**. https://doi.org/10.1152/AJPHEART.00392.2012

26. Morse JC, Huang J, Khona N, Miller EJ, Siwik DA, Colucci WS, et al. Up-regulation of Intracellular Calcium Handling Underlies the Recovery of Endotoxemic Cardiomyopathy in Mice. *Anesthesiology* 2017;**126**:1125–1138. https://doi.org/10.1097/ALN.0000000000001627

27. Goodman JB, Qin F, Morgan RJ, Chambers JM, Croteau D, Siwik DA, et al. Redox-Resistant SERCA [Sarco(endo)plasmic Reticulum Calcium ATPase] Attenuates Oxidant-Stimulated Mitochondrial Calcium and Apoptosis in Cardiac Myocytes and Pressure Overload-Induced Myocardial Failure in Mice. *Circulation* 2020;**142**:2459–2469. https://doi.org/10.1161/CIRCULATIONAHA.120.048183

28. Lopes M, Brejchova K, Riecan M, Novakova M, Rossmeisl M, Cajka T, et al. Metabolomics atlas of oral 13C-glucose tolerance test in mice. *Cell Rep* 2021;**37**. https://doi.org/10.1016/J.CELREP.2021.109833

29. Odriozola CP, Cordero JÁG, Daura J, Sanz M, Martínez-Blanes JM, Avilés MÁ. Reliability of blood lactate as a measure of exercise intensity in different strains of mice during forced treadmill running. *PLoS One* 2019;**14**. https://doi.org/10.1371/JOURNAL.PONE.0215584

30. Ari C, Murdun C, Goldhagen C, Koutnik AP, Bharwani SR, Diamond DM, et al. Exogenous Ketone Supplements Improved Motor Performance in Preclinical Rodent Models. *Nutrients* 2020;**12**:1–18. https://doi.org/10.3390/NU12082459

31. Toepfer CN, Garfinkel AC, Venturini G, Wakimoto H, Repetti G, Alamo L, et al. Myosin Sequestration Regulates Sarcomere Function, Cardiomyocyte Energetics, and Metabolism, Informing the Pathogenesis of Hypertrophic Cardiomyopathy. *Circulation* 2020;**141**:828–842. https://doi.org/10.1161/CIRCULATIONAHA.119.042339

32. Michas C, Karakan MÇ, Nautiyal P, Seidman JG, Seidman CE, Agarwal A, et al. Engineering a living cardiac pump on a chip using high-precision fabrication. *Sci Adv* 2022;**8**. https://doi.org/10.1126/SCIADV.ABM3791

33. Silva dos Santos D, Turaça LT, Coutinho KC da S, Barbosa RAQ, Polidoro JZ, Kasai-Brunswick TH, et al. Empagliflozin reduces arrhythmogenic effects in rat neonatal and human iPSC-derived cardiomyocytes and improves cytosolic calcium handling at least partially independent of NHE1. *Sci Rep* 2023;**13**. https://doi.org/10.1038/S41598-023-35944-5

34. Scheen AJ. Pharmacokinetic and pharmacodynamic profile of empagliflozin, a sodium glucose co-transporter 2 inhibitor. *Clin Pharmacokinet* 2014;**53**:213–225. https://doi.org/10.1007/S40262-013-0126-X

35. Hong R, Koga Y, Bandyadka S, Leshchyk A, Wang Y, Akavoor V, et al. Comprehensive generation, visualization, and reporting of quality control metrics for single-cell RNA sequencing data. *Nat Commun* 2022;**13**. https://doi.org/10.1038/S41467-022-29212-9

36. Chakraborty A, Kim A, AlAbdullatif S, Campbell J, Alekseyev Y, Kaplan U, et al. Endothelial Erg Regulates Expression of Pulmonary Lymphatic Junctional and Inflammation Genes in Mouse Lungs Impacting Lymphatic Transport. *Res Sq* 2024. https://doi.org/10.21203/RS.3.RS-3808970/V1

37. Phan BDN, Ray MH, Xue X, Fu C, Fenster RJ, Kohut SJ, et al. Single nuclei transcriptomics in human and non-human primate striatum in opioid use disorder. *Nat*

*Commun* 2024;**15**. https://doi.org/10.1038/S41467-024-45165-7

38. Sun Y, Benmhammed H, Abdullatif S Al, Habara A, Fu E, Brady J, et al. PGC-1α agonism induces fetal hemoglobin and exerts antisickling effects in sickle cell disease. *Sci Adv* 2024;**10**. https://doi.org/10.1126/SCIADV.ADN8750

39. Blum BC, Emili A. Omics Notebook: robust, reproducible and flexible automated multiomics exploratory analysis and reporting. *Bioinforma Adv* 2021;**1**:vbab024. https://doi.org/10.1093/BIOADV/VBAB024

40. Korotkevich G, Sukhov V, Budin N, Shpak B, Artyomov MN, Sergushichev A. Fast gene set enrichment analysis. *bioRxiv* 2021. https://doi.org/10.1101/060012

41. Blighe K, Rana S, Lewis M. EnhancedVolcano: publication-ready volcano plots with enhanced colouring and labeling. Available from: https://bioconductor.org/packages/devel/bioc/vignettes/EnhancedVolcano/inst/doc/EnhancedVolcano.html (accessed 19 June 2024)

42. Marian AJ, Braunwald E. Hypertrophic Cardiomyopathy: Genetics, Pathogenesis, Clinical Manifestations, Diagnosis, and Therapy. *Circ Res* 2017;**121**:749–770. https://doi.org/10.1161/CIRCRESAHA.117.311059

43. Wu Q, Yao Q, Hu T, Yu J, Jiang K, Wan Y, et al. Dapagliflozin protects against chronic heart failure in mice by inhibiting macrophage-mediated inflammation, independent of SGLT2. *Cell Rep Med* 2023;**4**:101334. https://doi.org/10.1016/J.XCRM.2023.101334

44. Withaar C, Meems LMG, Markousis-Mavrogenis G, Boogerd CJ, Silljé HHW, Schouten EM, et al. The effects of liraglutide and dapagliflozin on cardiac function and structure in a multi-hit mouse model of heart failure with preserved ejection fraction. *Cardiovasc Res* 2021;**117**:2108–2124. https://doi.org/10.1093/CVR/CVAA256

45. Li X, Lu Q, Qiu Y, Carmo JM Do, Wang Z, Silva AA da, et al. Direct Cardiac Actions of the Sodium Glucose Co-Transporter 2 Inhibitor Empagliflozin Improve Myocardial Oxidative Phosphorylation and Attenuate Pressure-Overload Heart Failure. *J Am Heart Assoc* 2021;**10**:e018298. https://doi.org/10.1161/JAHA.120.018298

46. Salah HM, Verma S, Santos-Gallego CG, Bhatt AS, Vaduganathan M, Khan MS, et al. Sodium-Glucose Cotransporter 2 Inhibitors and Cardiac Remodeling. *J Cardiovasc Transl Res* 2022;**15**:944–956. https://doi.org/10.1007/S12265-022-10220-5

47. Fang L, Ellims AH, Beale AL, Taylor AJ, Murphy A, Dart AM. Systemic inflammation is associated with myocardial fibrosis, diastolic dysfunction, and cardiac hypertrophy in patients with hypertrophic cardiomyopathy. *Am J Transl Res* 2017;**9**:5063–5073.

48. Lillo R, Graziani F, Franceschi F, Iannaccone G, Massetti M, Olivotto I, et al. Inflammation across the spectrum of hypertrophic cardiac phenotypes. *Heart Fail Rev* 2023;**28**:1065–1075. https://doi.org/10.1007/S10741-023-10307-4

49. Alsereidi FR, Khashim Z, Marzook H, Gupta A, Al-Rawi AM, Ramadan MM, et al. Targeting inflammatory signaling pathways with SGLT2 inhibitors: Insights into cardiovascular health and cardiac cell improvement. *Curr Probl Cardiol* 2024;**49**:102524. https://doi.org/10.1016/J.CPCARDIOL.2024.102524

50. Su S, Ji X, Li T, Teng Y, Wang B, Han X, et al. The changes of cardiac energy metabolism with sodium-glucose transporter 2 inhibitor therapy. *Front Cardiovasc Med* 2023;**10**:1291450. https://doi.org/10.3389/FCVM.2023.1291450
